# Supplementary material for: Reshuffling of the ancestral core-eudicot genome shaped chromatin topology and epigenetic modification in Panax
Source: Nat Commun. 2022 Apr 7;13:1902. doi: 10.1038/s41467-022-29561-5 (PMC8989883; doi:10.1038/s41467-022-29561-5)
Supplement: Supplementary file 1 — Supplementary Information [file 41467_2022_29561_MOESM1_ESM.pdf]

Reshuffling of the ancestral core-eudicot genome shaped chromatin topology and epigenetic modification in *Panax*

Wang *et al.*

Supplementary Note 1. Plant materials, genome assembly and annotation

Samples of the *P. ginseng* (2n = *4x* = 48) and *P. quinquefolius* (2n = *4x* = 48) were collected from Jilin Province of China. Samples of the *P. stipuleanatus* (2n = *2x* = 24) and *P. japonicus* (2n = *4x* = 48) were collected from Yunnan Province of China and Hokkaido of Japan, respectively. Karyotype of the four species were checked with fluorescent in situ hybridization (Supplementary Figure 1). All samples used in this study were grown in greenhouse at Fudan University under the same conditions (25°C/12 hours, 16°C/12 hours).

The paired-end Illumina DNA libraries were constructed with an average insert length of 350 bp, and 262.71-302.73 Gb (44.53-66.42× coverage) sequencing data were generated from the four *Panax* species on the Illumina Novaseq platform (Illumina, CA, USA). Genome features of the four *Panax* species were surveyed by Jellyfish[1] based on the Illumina short reads (Supplementary Figure 2). The diploid species *P. stipuleanatus* (genome size = 2.15 Gb, heterozygous rate = 0.94%) possesses a small genome that is comparable to the tetraploid *P. japonicus* (genome size = 2.09 Gb, heterozygous rate = 1.07%). In contrast, both the *P. ginseng* (genome size = 3.41 Gb, heterozygous rate = 0.11%) and *P. quinquefolius* (genome size = 3.60 Gb, heterozygous rate = 0.04%), possess larger genome but with lower level of heterozygosity. Genome sizes of the four species were also calculated based on flow cytometry with three biological replicates (Supplementary Figure 3). Genome sizes of the three species, *P. stipuleanatus* (genome size = 1.79-1.81 Gb), *P. japonicus* (genome size = 1.90-1.93 Gb) and *P. ginseng* (3.34-3.35 Gb) are comparable to those of estimated by genome survey. However, genome size of the *P. quinquefolius* calculated by flow cytometry (genome size = 4.13-4.14 Gb) is larger than that of estimated based on genome survey (genome size = 3.60 Gb). Our genome collinearity analyses showed that, while the four species varied dramatically in genome size, they still maintained high collinearity across the 12 orthologous chromosomes (Supplementary Figure 4).

Taxonomic positions of the four species used in this study were consistent with previous studies[2], with the three tetraploid species (*P. ginseng*, *P. quinquefolius* and *P. japonicus*) clustering as a clade and the early-diverging species *P. stipuleanatus* forming a distinct clade (Supplementary Figure 5). Notably, our samples of the *P. japonicus* are phylogenetically distinct to the other subspecies/varieties that are naturally distributed in China. It confirms the independent taxonomical position of this Japanese species. Polyploidization history of the *P. japonicus* based on Ks value also confirmed its tetraploid karyotype (Supplementary Figure 6).

Based on the genome features estimated above, three different strategies were employed to *de novo* assemble the reference genomes of the four *Panax* species. For the *P. stipuleanatus* and *P. japonicus* with smaller genome size, a total of 218.15-247.19 Gb long reads were produced using PacBio Sequel platform (PacBio, CA, USA). Then, the long sequence consensus was generated by FALCON[3] and polished by Illumina short reads using Pilon[4]. High quality sequence consensus was further extended by the linked-reads generated by 10xGenomics. We then scaffolded the reference genome to chromosome-level by using Hi-C reads and performed gap-filling with pre-assembled PacBio reads. For the *P. ginseng* and *P. quinquefolius* with larger genome size, 257.47-297.63 Gb high quality long reads were obtained from Nanopore platform (Oxford Nanopore Technologies, Oxford, UK). The corrected long Nanopore reads of *P. quinquefolius* were used to assemble reference genome using the program SMARTdenovo[5]. Reference genome of the *P. ginseng* was assembled with the corrected long Nanopore reads using Nextdenovo (https://github.com/Nextomics/NextDenovo). The draft assemblies of the four species were polished by corrected long reads using Racon[6] and Illumina short reads using Pilon[4] separately. The Hi-C data was used to link the corrected sequence contigs into pseudochromosomes using LACHESIS[7]. Broadly consistent with the above estimated genome sizes, total lengths of the *P. stipuleanatus* (genome size = 1.96 Gb) and *P. japonicus* (genome size = 2.02 Gb) were smaller than those of *P. ginseng* (genome size = 3.36 Gb) and *P. quinquefolius* (genome size = 3.57 Gb) (Table 1). Annotation of the protein-coding genes of the four species were performed using the programs Genscan[8], Augustus[9], GlimmerHMM[10], GeneID[11] and SNAP[12]. Gene homeolog was predicted using GeMoMa[13]. All the protein-coding genes annotated by distinct strategies were combined by EVM[14].

Supplementary Note 2. Quality control of the genome assemblies

Quality control of the four genome assemblies were performed for the DNA-based pseudomolecules, protein genes and repeat sequences, independently. Firstly, short Illumina reads generated from the four species were mapped onto their own assembled genomes using BWA[15]. About 98.36-99.67% of the Illumina reads were successfully mapped back to the genome assemblies of the four species. It suggests that the four newly genome assemblies are highly representative. Secondly, genome completeness and contiguity were estimated using Benchmarking universal single copy orthologs (BUSCO) and LTR assembly index (LAI). All the four species possessed high completeness of the protein-coding genes (BUSCO = 93.10-95.14%) (Table 1), which are comparable to previously assembled *Panax* genomes (BUSCO = 82.4-96.6%) [16-19]. Likewise, the four newly assembled genomes also showed high level of contiguity of the repeat sequence (LAI = 7.13-16.24). Thirdly, we performed genome collinear analyses between the four newly assembled *Panax* genomes

and previously published genomes of the *P. notoginseng* (within the same genus *Panax*)[19]and *Eleutherococcus senticosus* (within the same family Araliaceae)[20]. Our comparisons revealed high level of genome collinearity among these genomes (Supplementary Figures 4, and 8-18). It is notable that our comparisons identified six large fragment translocations and conversions, three of which are specific to the genus *Panax*. In brief, the conversion event I and translocation event II are supposed to occur after the divergence between *P. stipuleanatus* and the other four *Panax* species. The remaining three events (III, IV and V) are specific to the *P. notoginseng*. Notably, through comparing the genome collinearity between the genera *Panax* and *Eleutherococcus*, the event IV and V are more likely due to the low quality of the *P. notoginseng* genome. In addition, we also identified three chromosomal conversion events (VI, VII and VIII) that might have occurred after the divergence between the genera *Eleutherococcus* and *Panax* (Supplementary Figure 4, and 8-18).

To further validate the genome assemblies of the four species, we separated the two subgenomes of the three tetraploid *Panax* species based on the genome collinearity and sequence homoeology (Supplementary Table 1). Phylogenetic inference based on single copy orthologous genes confirmed that the subgenome A of the three tetraploid species clustered with *P. notoginseng* and the subgenome B formed a monophyletic clade (Supplementary Figure 5). We also performed electric fluorescence in situ hybridization (FISH) simulation with previously developed probes[21-23]. Our simulated karyotypes of the five *Panax* species (Supplementary Figure 44) are highly similar to previously inferred FISH karyotypes[22,23]. In particular, the two diploid and subgenome A of the three tetraploid species showed apparently stronger signal compared to the subgenome B of the three tetraploid species. Yet, we are not able to rule out the fact that there could still be mis-assemblies in each of the four newly assembled genomes. Independent assembly of the four *Panax* species should be able to minimize errors in the inference of ancestral karyotype evolution.

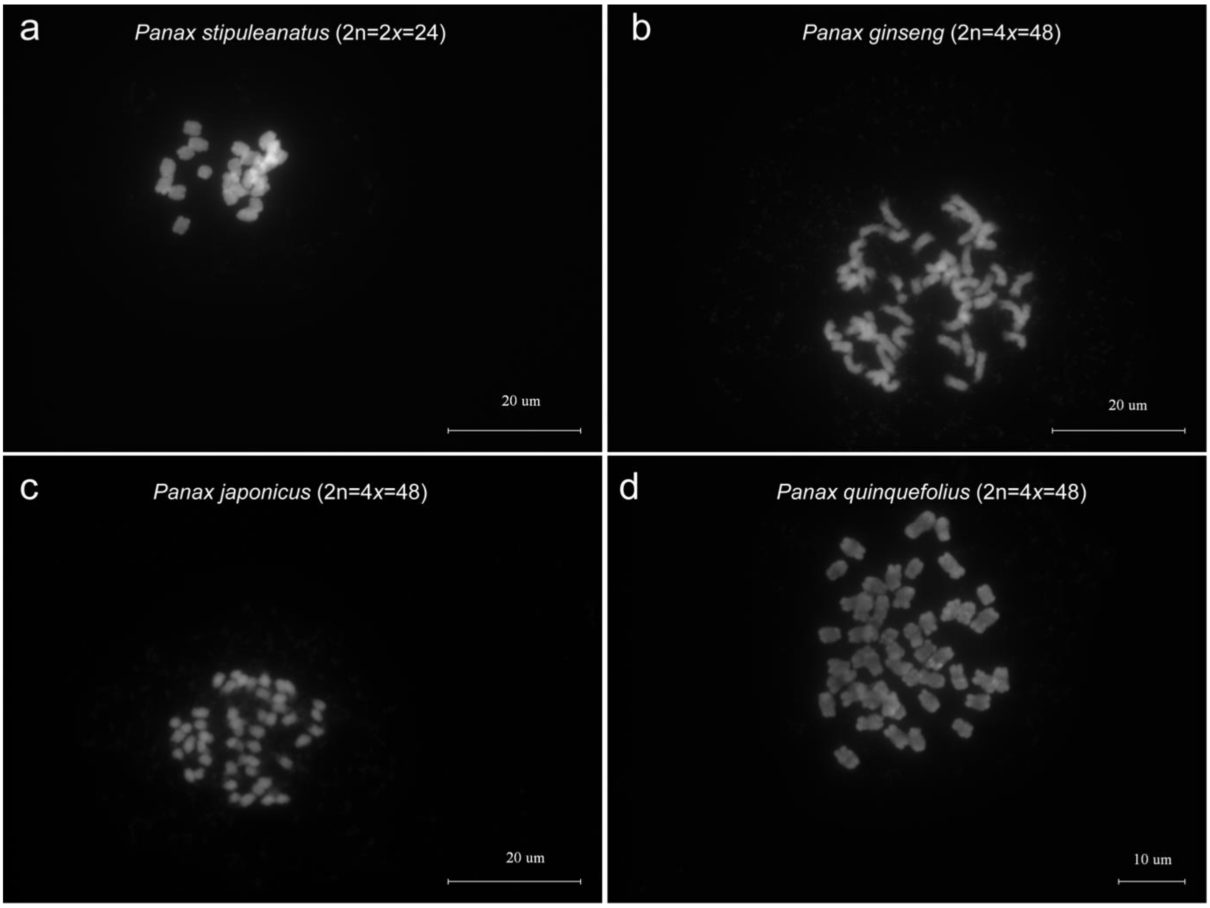

Supplementary Figure 1. Karyotype of the four *Panax* species used in this study. (a) Karyotype of the diploid species *Panax stipuleanatus* (2n=2*x*=24); (b-d) Karyotypes of the three tetraploid species *Panax ginseng*, *Panax japonicas* and *Panax quinquefolius*, respectively (2n=4*x*=48).

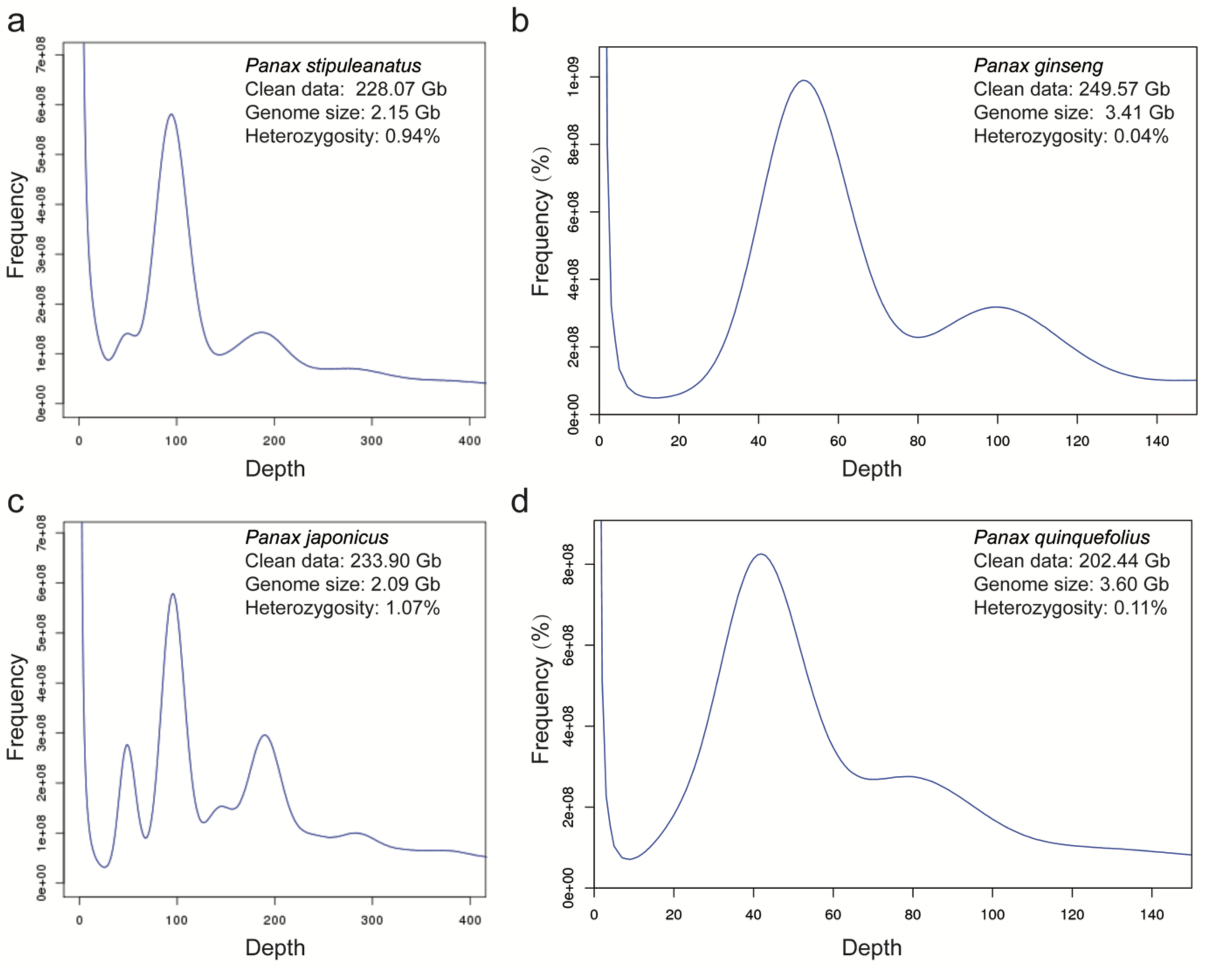

Supplementary Figure 2. Genome features of the four *Panax* species surveyed using Jellyfish[1] based on Illumina short reads. (a) Genome feature of the diploid species *Panax stipuleanatus* (2n=2*x*=24); (b-d) Genome features of the three tetraploid species *Panax ginseng*, *Panax japonicas* and *Panax quinquefolius*, respectively (2n=4*x*=48).

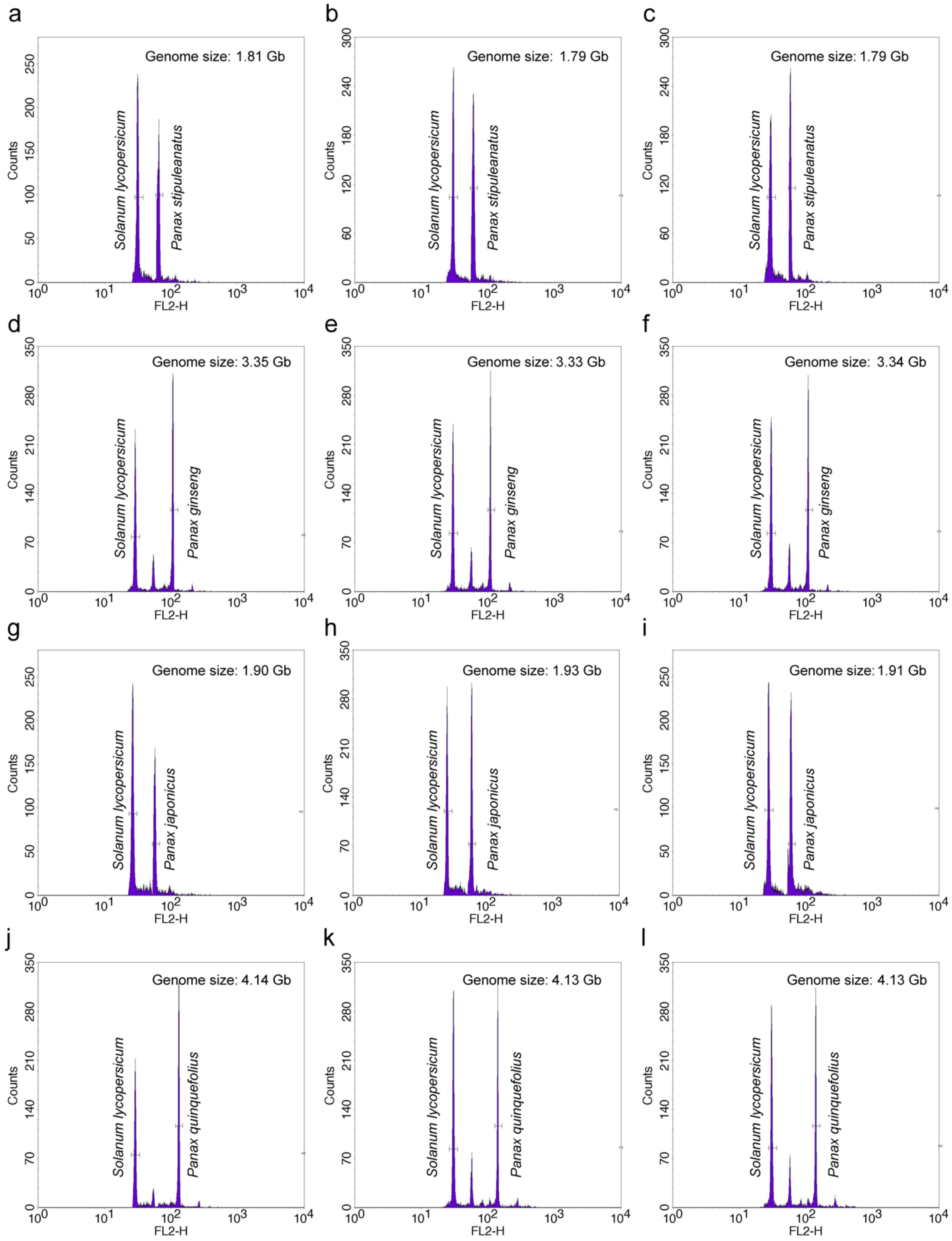

Supplementary Figure 3. Genome sizes of the four *Panax* species estimated by flow cytometry with three biological replicates. (a-c) *P. stipuleanatus*, (d-f) *P. japonicus*, (g-i) *P. ginseng*, (j-l) *P. quinquefolius*.

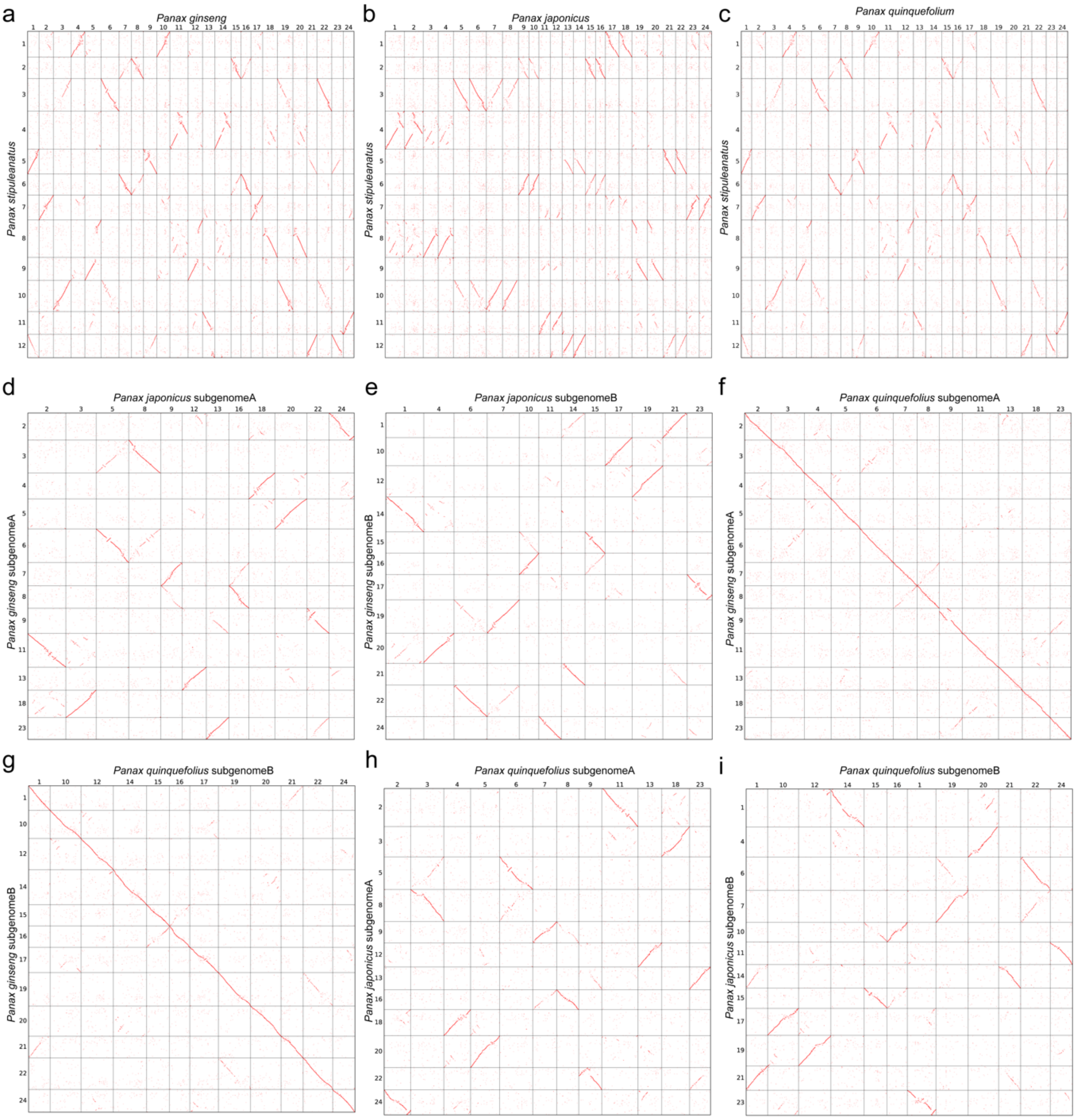

Supplementary Figure 4. Dotplot analyses of the genome collinearity between *Panax stipuleanatus* and the three tetraploid species (a-c) and between the two subgenomes among the three tetraploid species (d-i). Numbers on the x- and y-axis are the chromosome numbers for each species. Red lines indicate the collinear genomic regions on each chromosome.

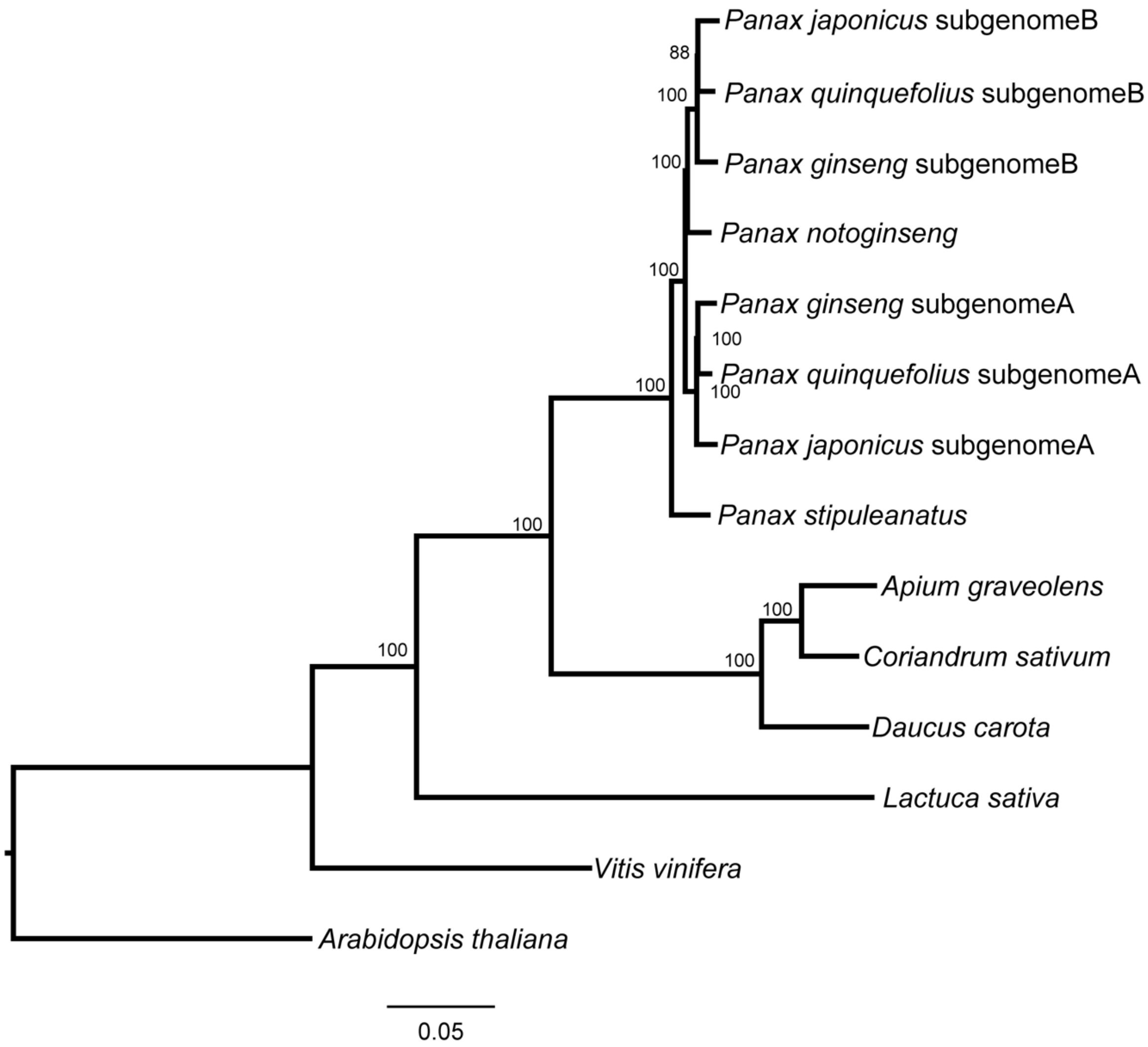

Supplementary Figure 5. Maximum likelihood (ML) tree of the five *Panax* species and six outgroup species based on single copy orthologous genes. Numbers on each branch denote the bootstrap support values. Subgenomes of the three tetraploid species are the same as Supplementary Table 1. Source data are provided as a Source Data file.

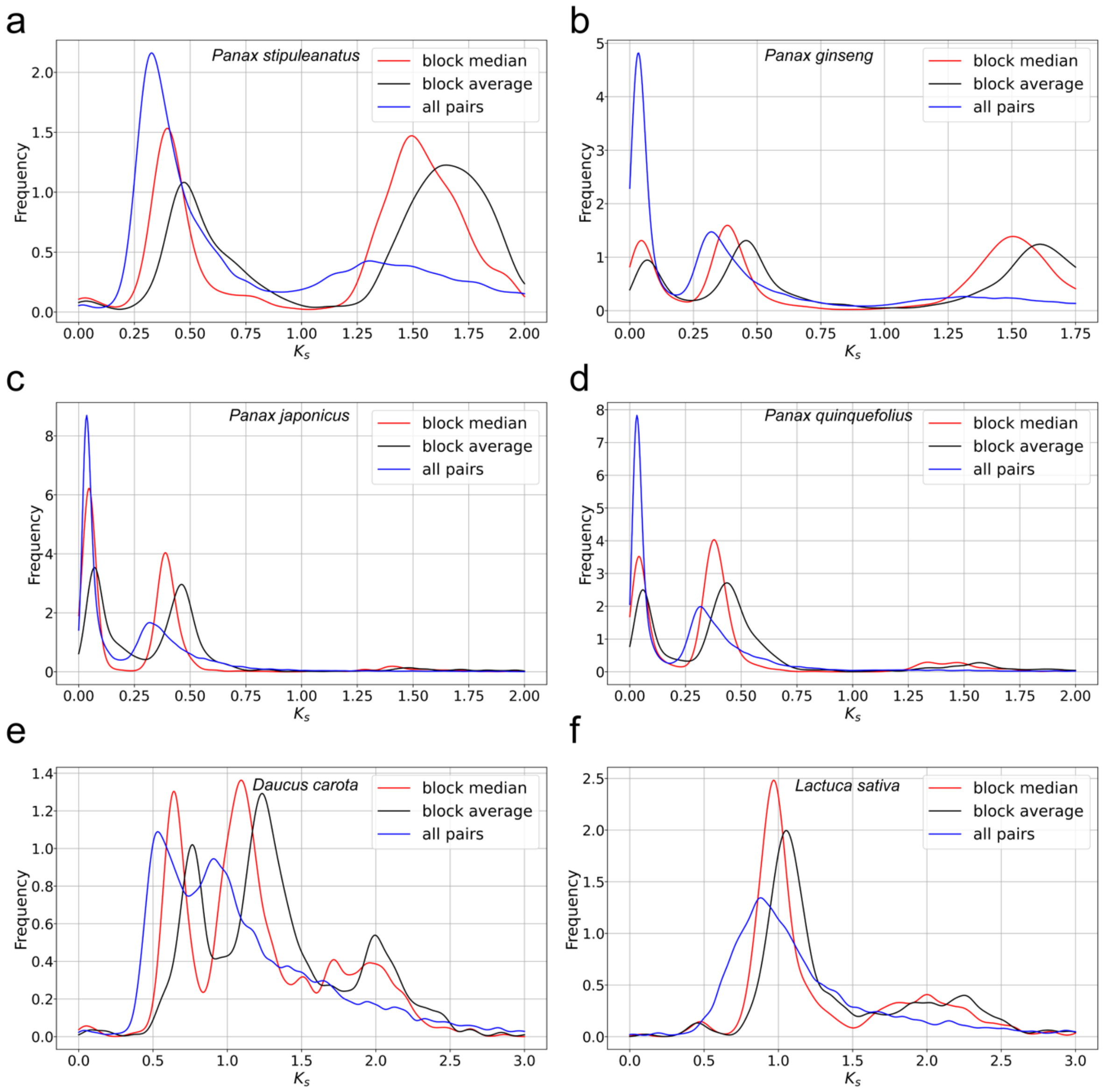

Supplementary Figure 6. Inference of the polyploidization histories in the six selected eudicot species. (a-d) Ks distribution patterns of the four *Panax* species (*P. stipuleanatus*, *P. ginseng*, *P. japonicus*, and *P. quinquefolius*). (e-f) Ks distribution patterns of the *Daucus carota and Lactuca sativa*. Block median and block average were estimated based on the median and average synonymous mutation rate (Ks) for each collinear block. All pairs were calculated based on paired collinear genes. Source data are provided as a Source Data file.

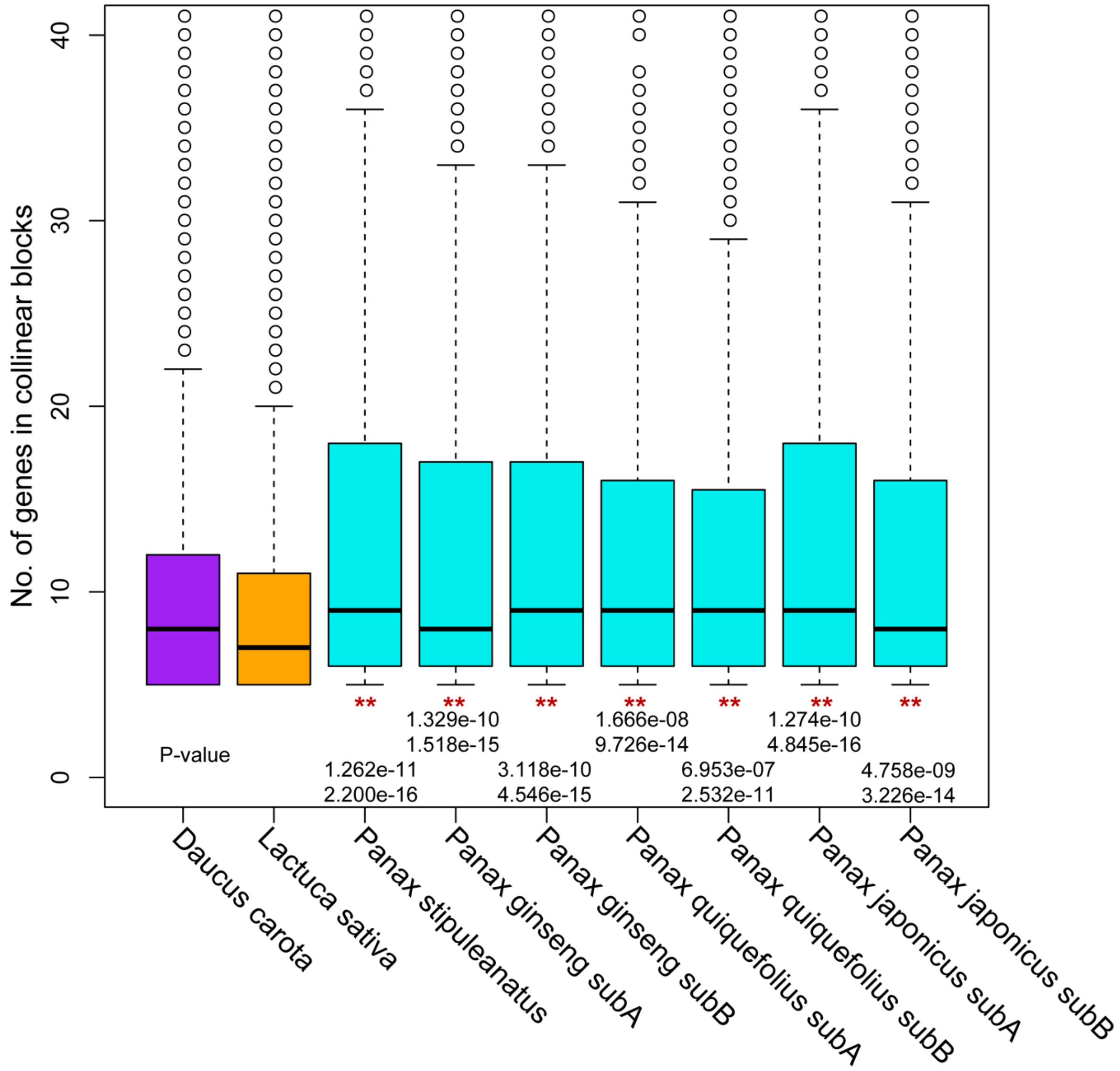

Supplementary Figure 7. Boxplot of the number of collinear genes between grape and the other six selected species (carrot, lettuce and ginseng species). Comparisons were performed between the seven *Panax* genomes and carrot and lettuce, respectively. The lower and upper whiskers of each colored box are the lowest and highest number of collinear genes, respectively. The black solid lines within each box are media values. The circles are extreme values. Lengths of the boxes are the interquartile range. All ancestral genes identified in the 42 genomic regions were used to the statistical analyses. **, two sides t-test, *p* values are shown at the bottom of each box. Source data are provided as a Source Data file.

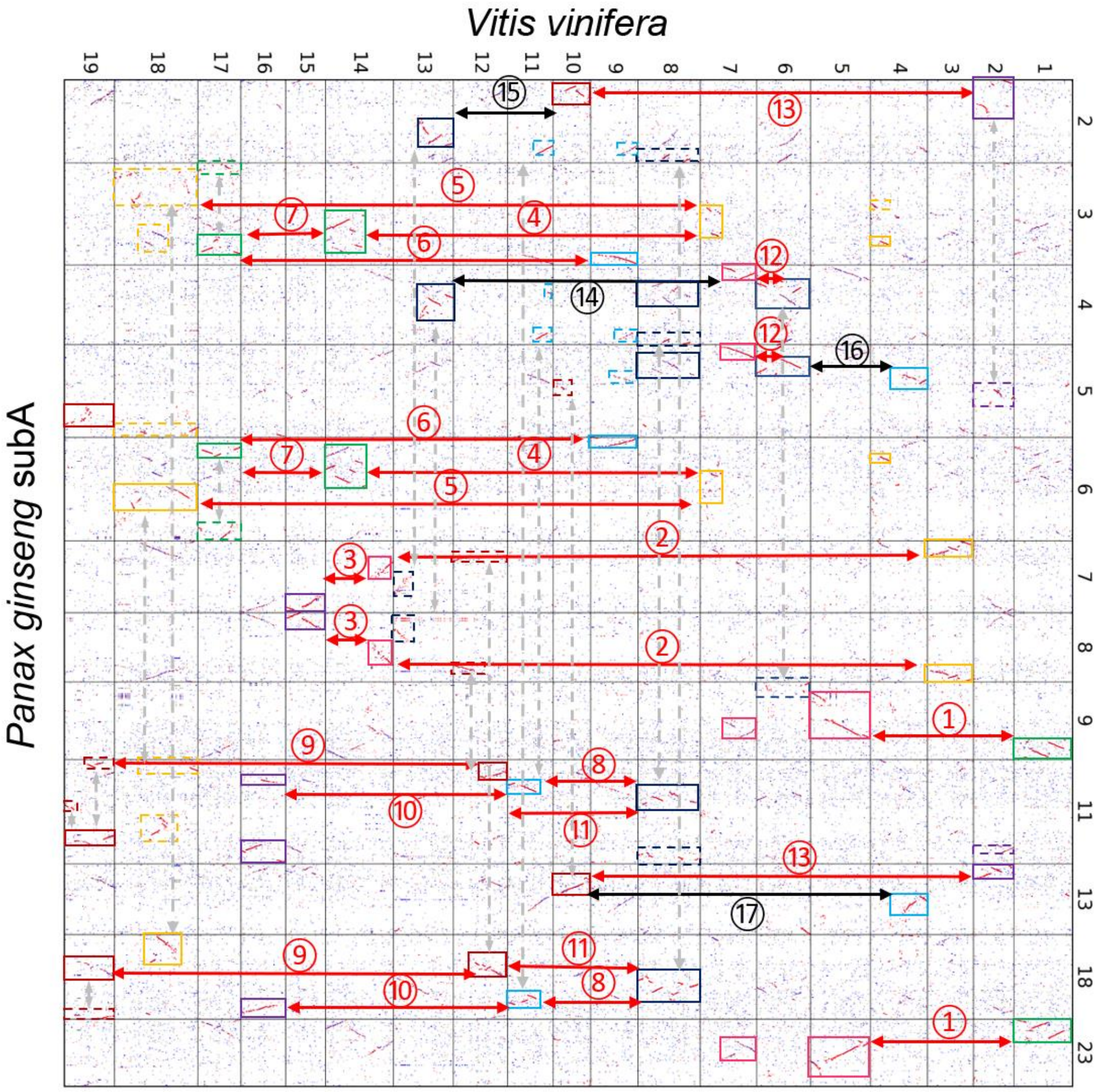

Supplementary Figure 8. Dotplot analyses of genome collinearity between grape (putative post-γ karyotype) and selected core-eudicot species. The two subgenomes of the three *Panax* tetraploid species are shown independently. Genomic regions within each colored box represent the ancestral core-eudicot chromosomes. Colors of each box are the same as the seven ancestral core-eudicot chromosomes in Figure 1. Red and black arrows indicate the 26 post-γ and four post-Pg-β chromosomal fusion events among the duplicated ancestral core-eudicot chromosomes, respectively. Twenty and ten of the post-γ chromosomal fusion events in *Panax* were also identified in carrot and lettuce genomes, respectively. Gray dashed arrows indicated the rearrangement of ancestral core-eudicot chromosomes in extant eudicot species.

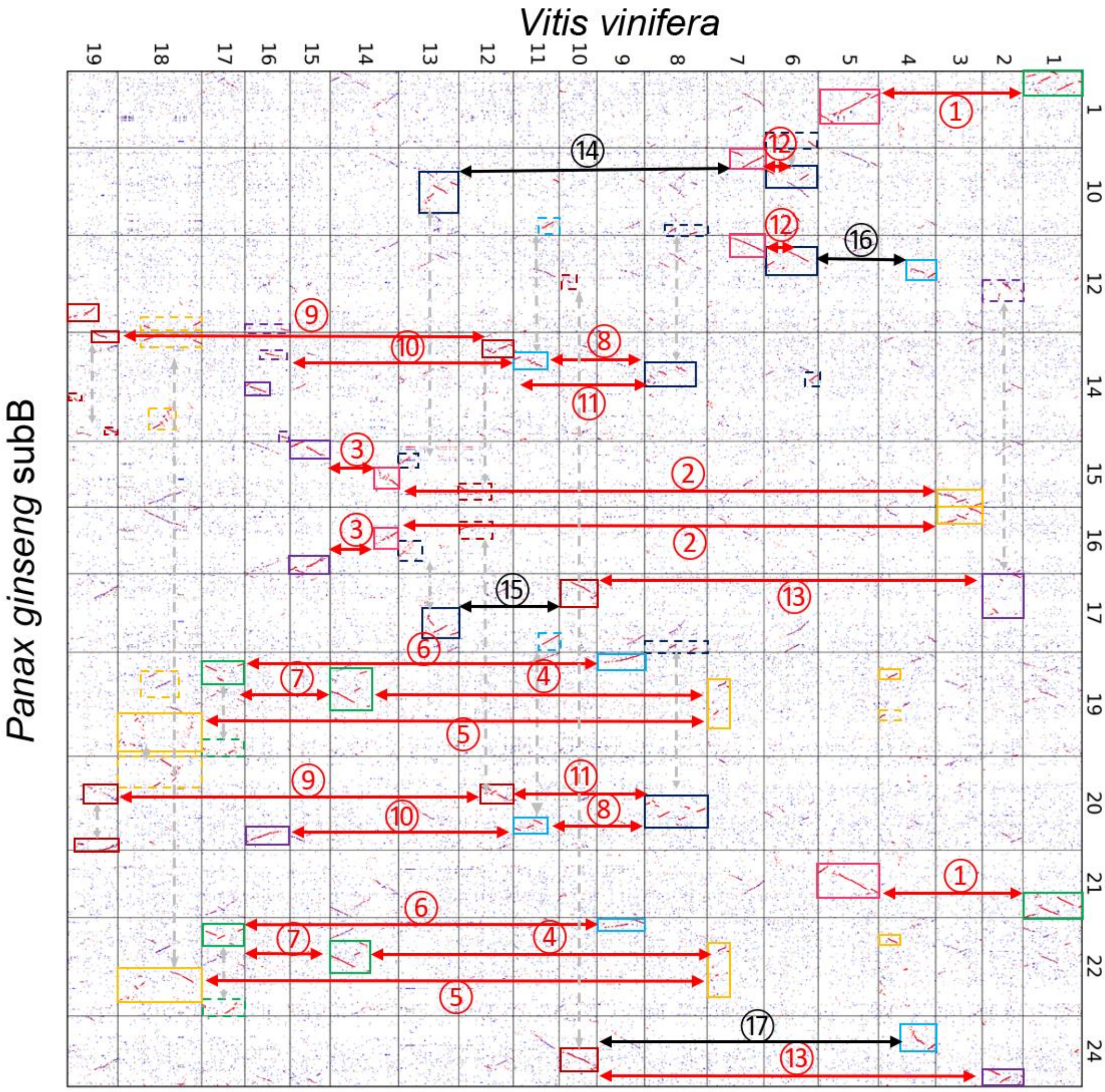

Supplementary Figure 9. Dotplot analyses of genome collinearity between grape (putative post-γ karyotype) and selected eudicot species. Interpretation is as in Supplementary Figure 8.

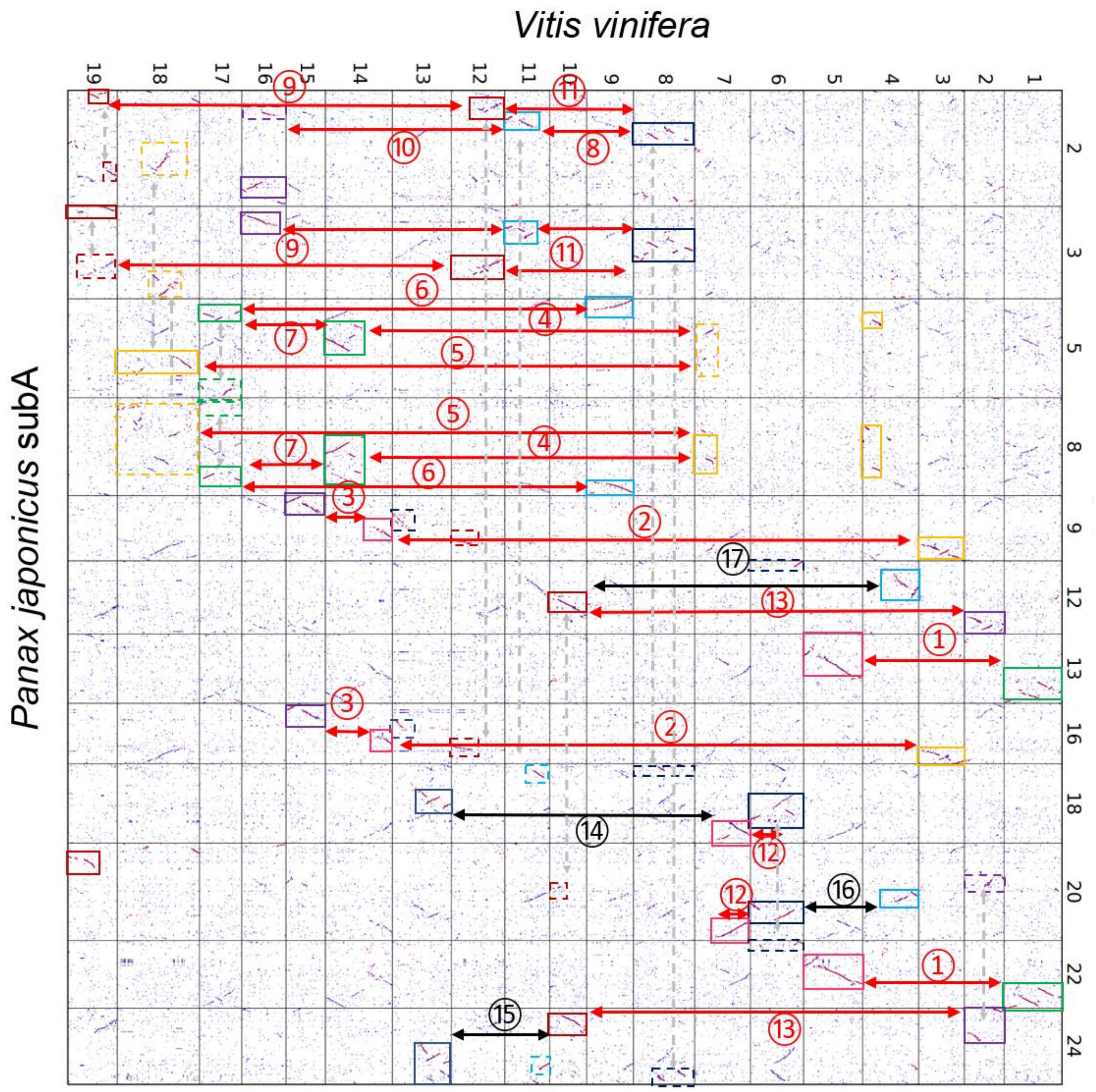

Supplementary Figure 10. Dotplot analyses of genome collinearity between grape (putative post-γ karyotype) and selected eudicot species. Interpretation is as in Supplementary Figure 8.

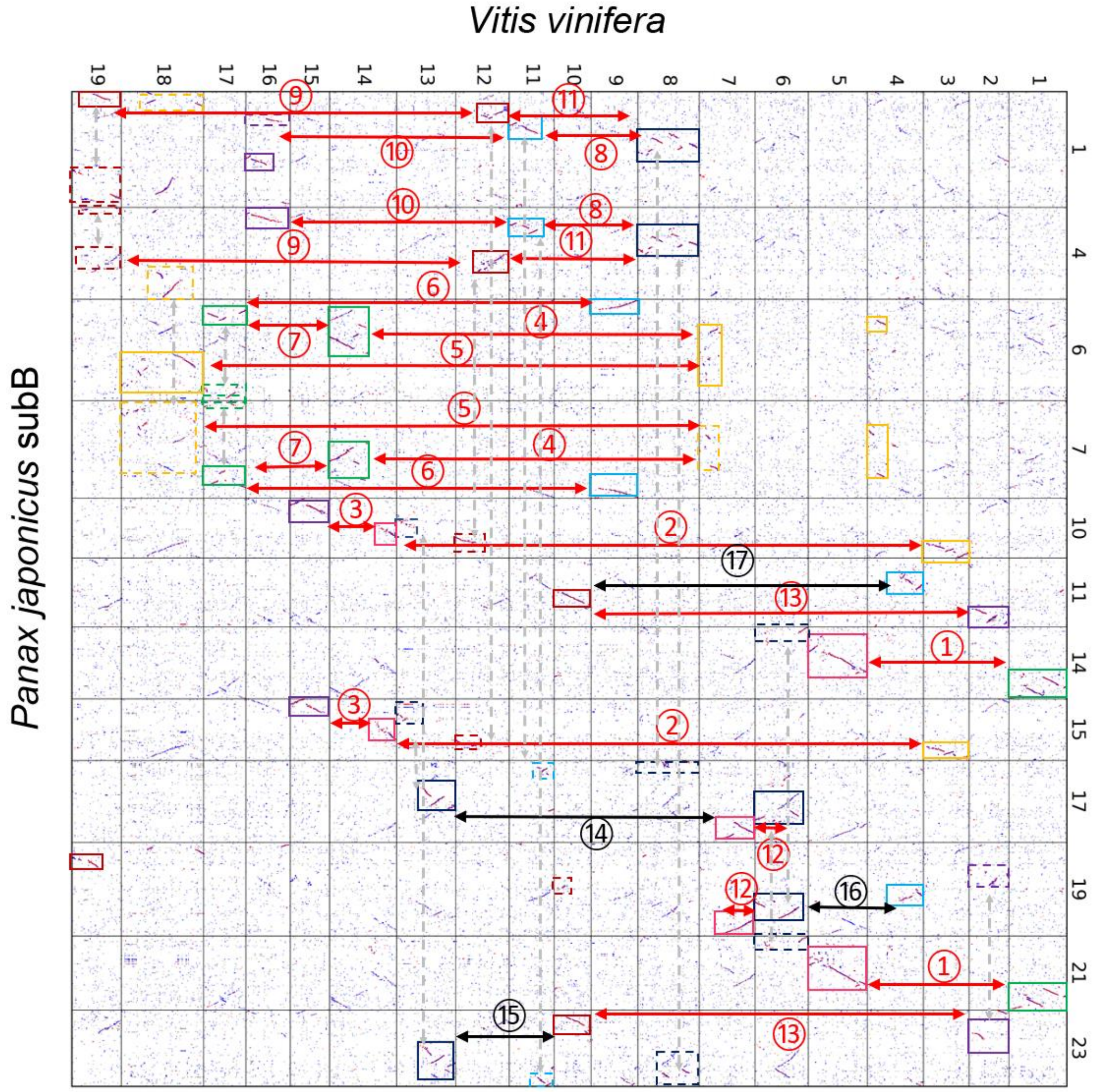

Supplementary Figure 11. Dotplot analyses of genome collinearity between grape (putative post-γ karyotype) and selected eudicot species. Interpretation is as in Supplementary Figure 8.

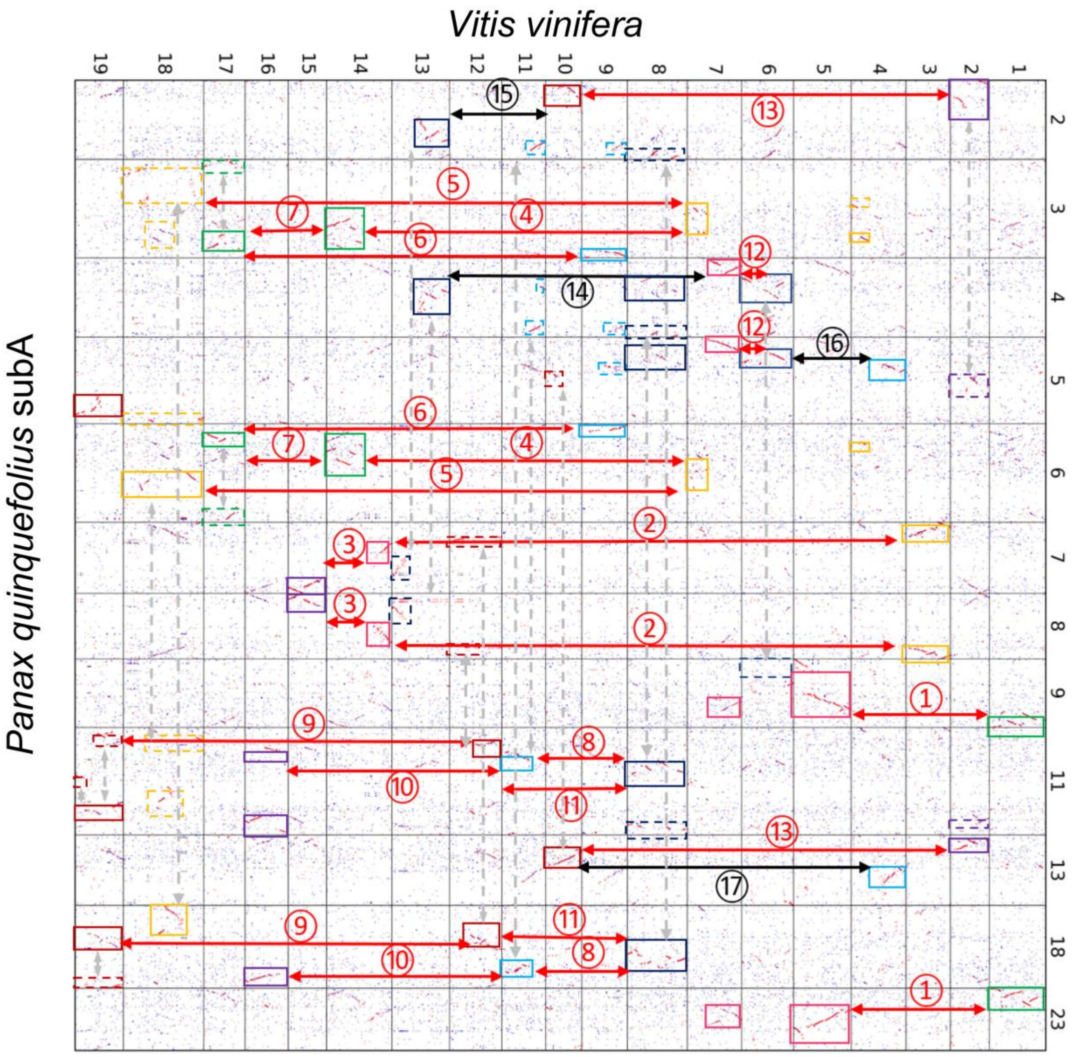

Supplementary Figure 12. Dotplot analyses of genome collinearity between grape (putative post-γ karyotype) and selected eudicot species. Interpretation is as in Supplementary Figure 8.

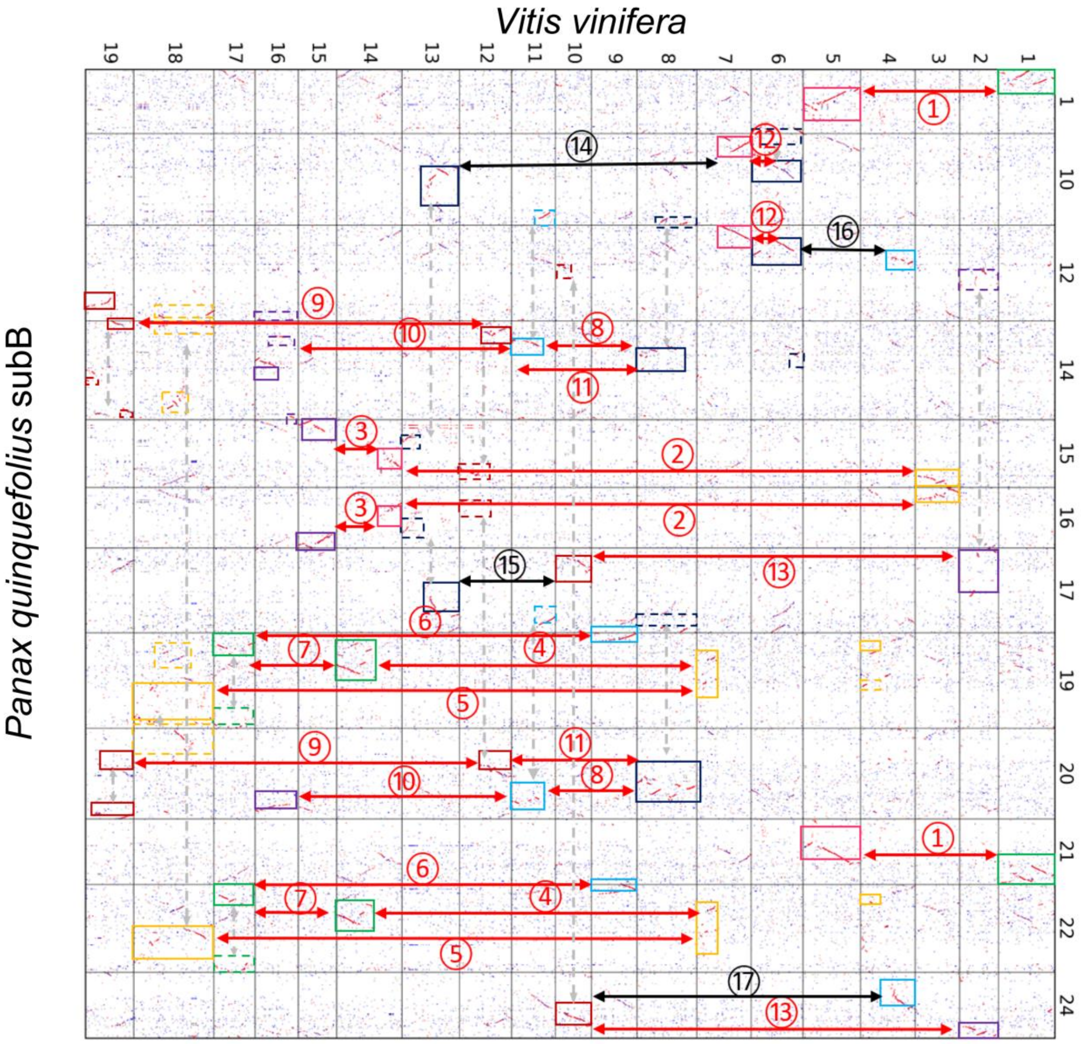

Supplementary Figure 13. Dotplot analyses of genome collinearity between grape (putative post-γ karyotype) and selected eudicot species. Interpretation is as in Supplementary Figure 8.

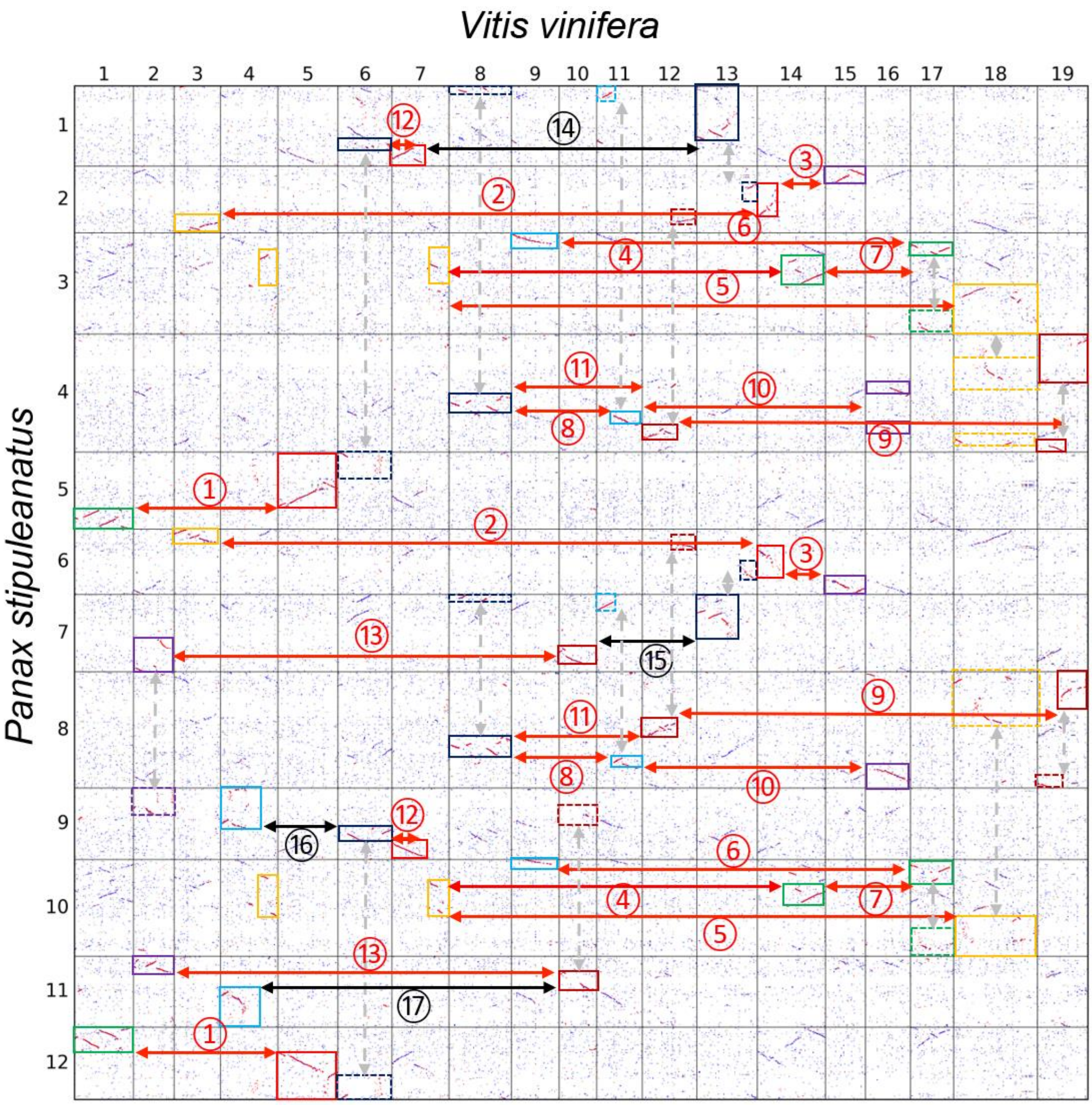

Supplementary Figure 14. Dotplot analyses of genome collinearity between grape (putative post-γ karyotype) and selected eudicot species. Interpretation is as in Supplementary Figure 8.

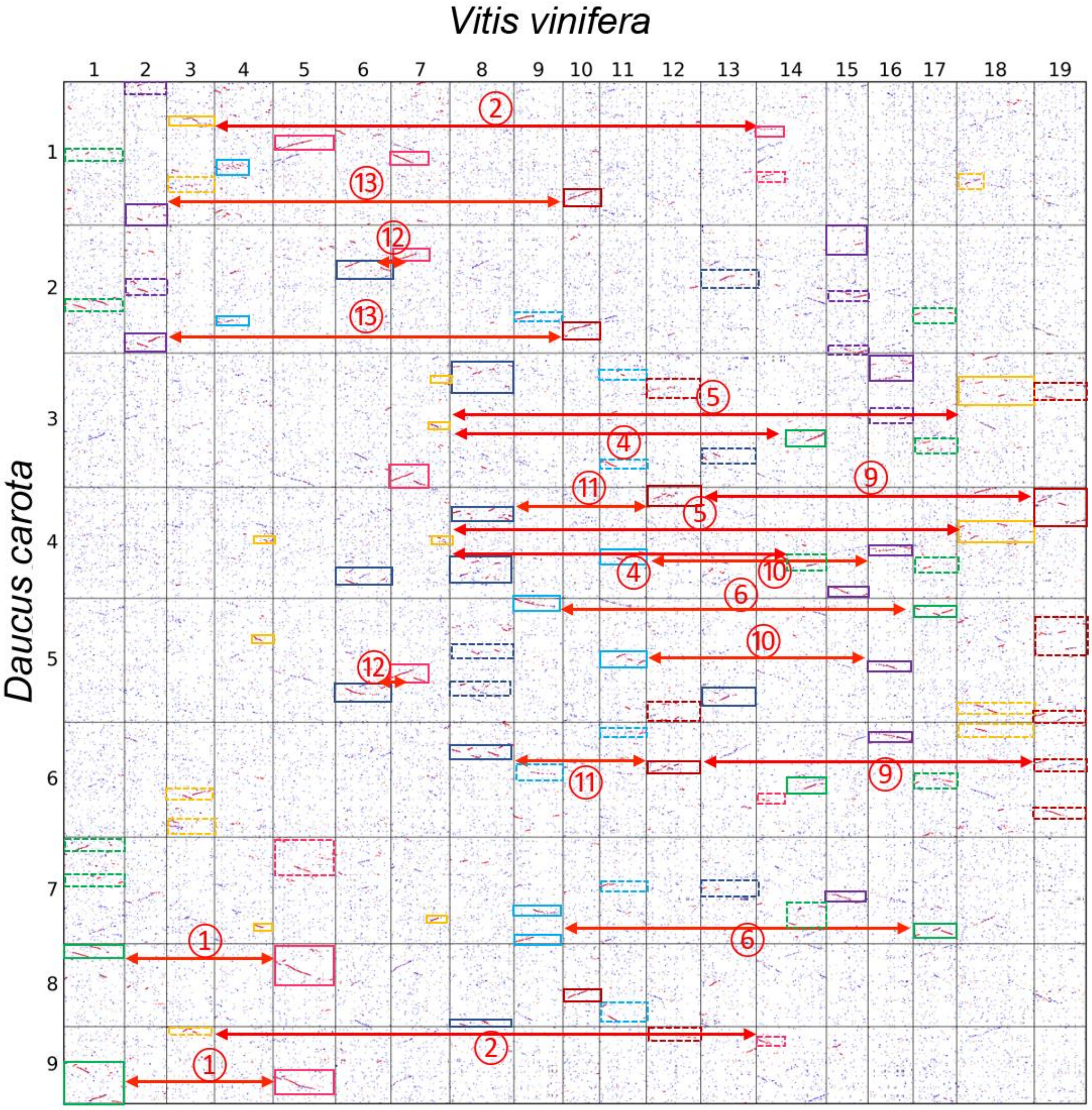

Supplementary Figure 15. Dotplot analyses of genome collinearity between grape (putative post-γ karyotype) and selected eudicot species. Interpretation is as in Supplementary Figure 8.

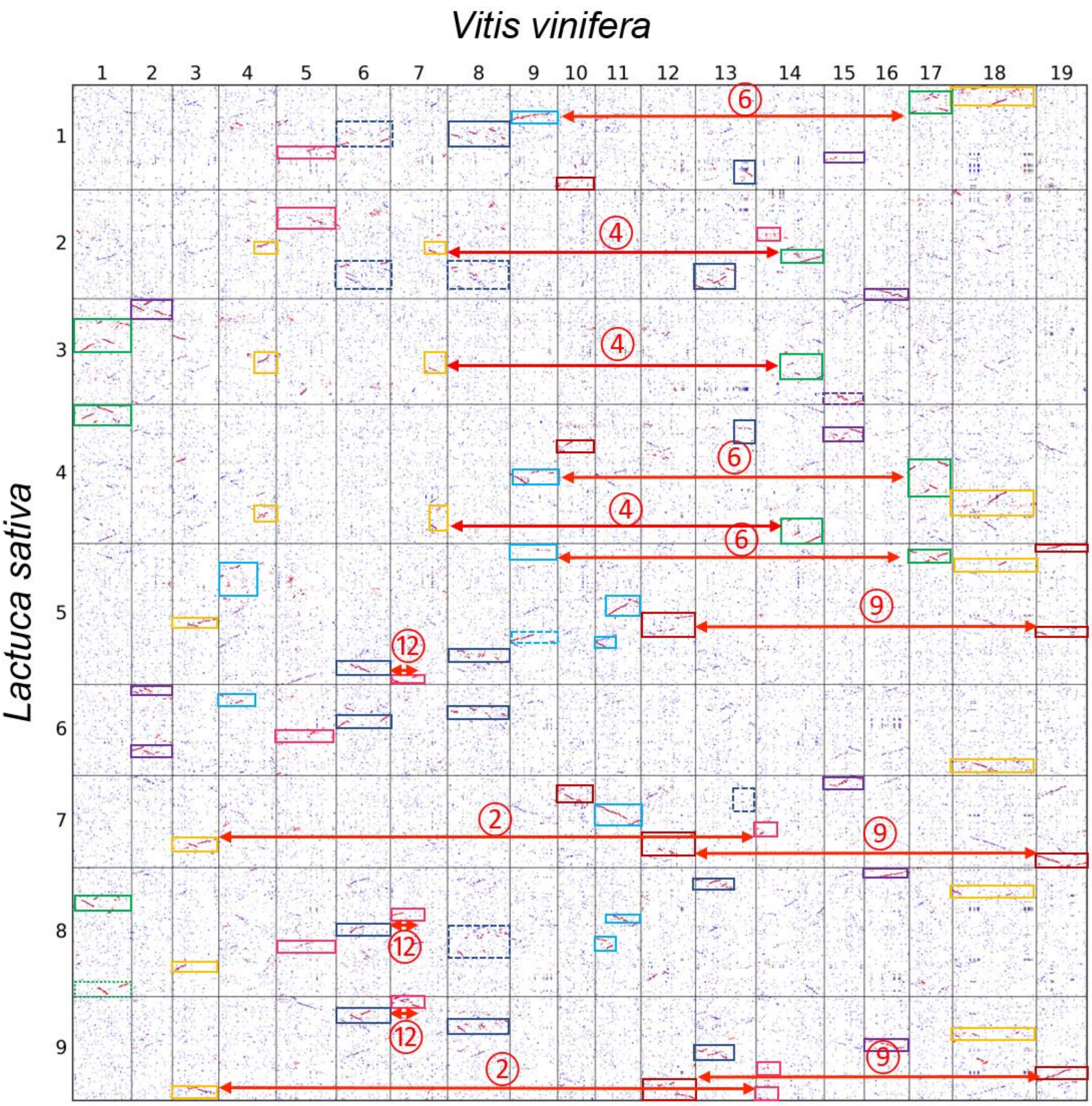

Supplementary Figure 16. Dotplot analyses of genome collinearity between grape (putative post-γ karyotype) and selected eudicot species. Interpretation is as in Supplementary Figure 8.

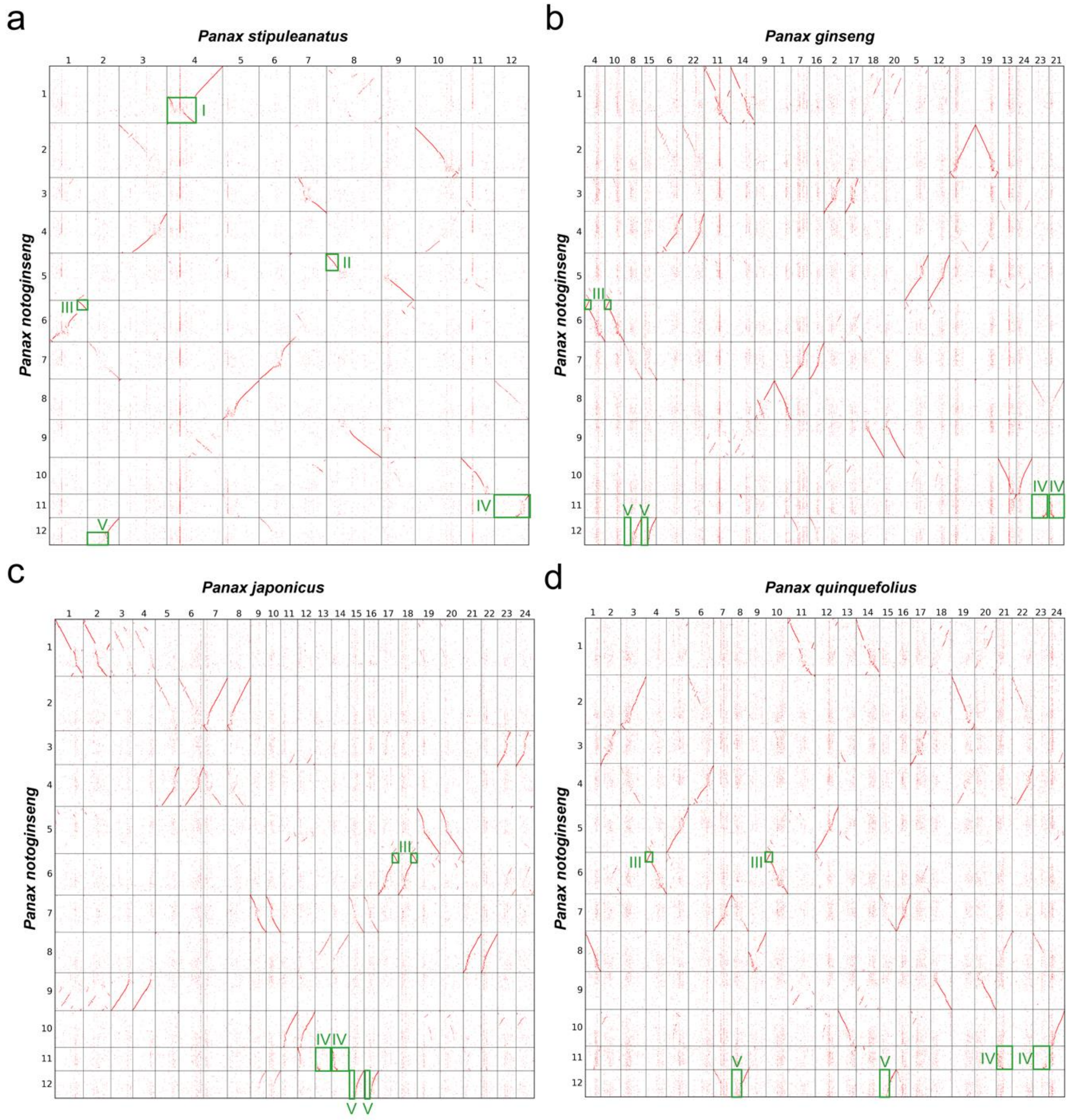

Supplementary Figure 17. Genome collinearity between the *Panax notoginseng* and the four newly assembled *Panax* species. (a) Comparison between *Panax notoginseng* and *Panax stipuleanatus*. (b) Comparison between *Panax notoginseng* and *Panax ginseng*; (c) Comparison between *Panax notoginseng* and *Panax japonicus*; (d) Comparison between *Panax notoginseng* and *Panax quinquefolius*. The five chromosomal rearrangement events (I-V) among these *Panax* species are indicated by green boxes.

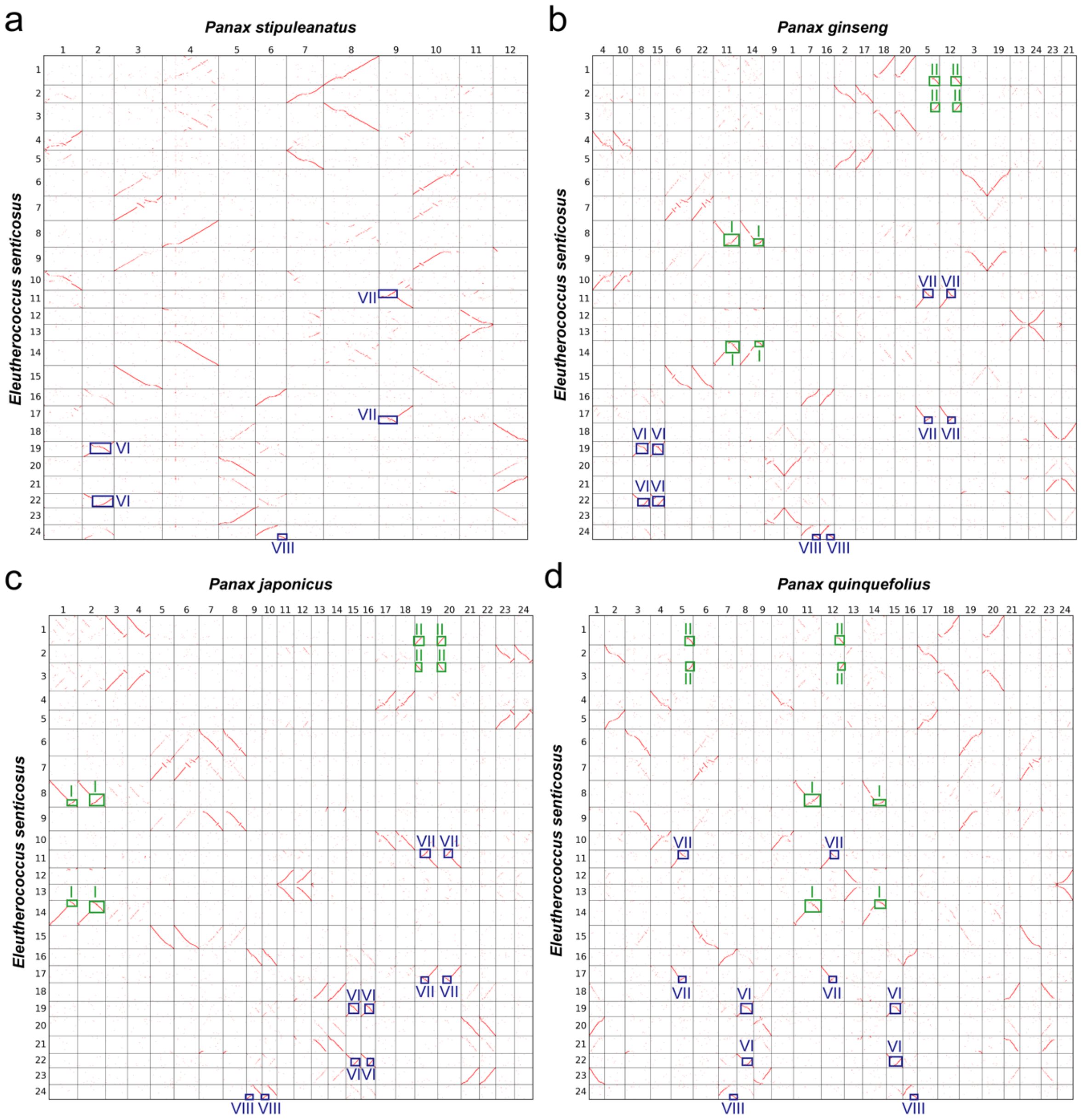

Supplementary Figure 18. Genome collinearity between the *Eleutherococcus senticosus* and our four newly assembled *Panax* species. (a) Comparison between *Eleutherococcus senticosus* and *Panax stipuleanatus*. (b) Comparison between *Eleutherococcus senticosus* and *Panax ginseng*; (c) Comparison between *Eleutherococcus senticosus* and *Panax japonicus*; (d) Comparison between *Eleutherococcus senticosus* and *Panax quinquefolius*. The five chromosomal rearrangement events between the *Eleutherococcus senticosus* and *Panax* species are shown with green (I and II) and blue (VI-VIII) boxes, respectively. The events I and II are the same as those shown in Supplementary Figure 17.

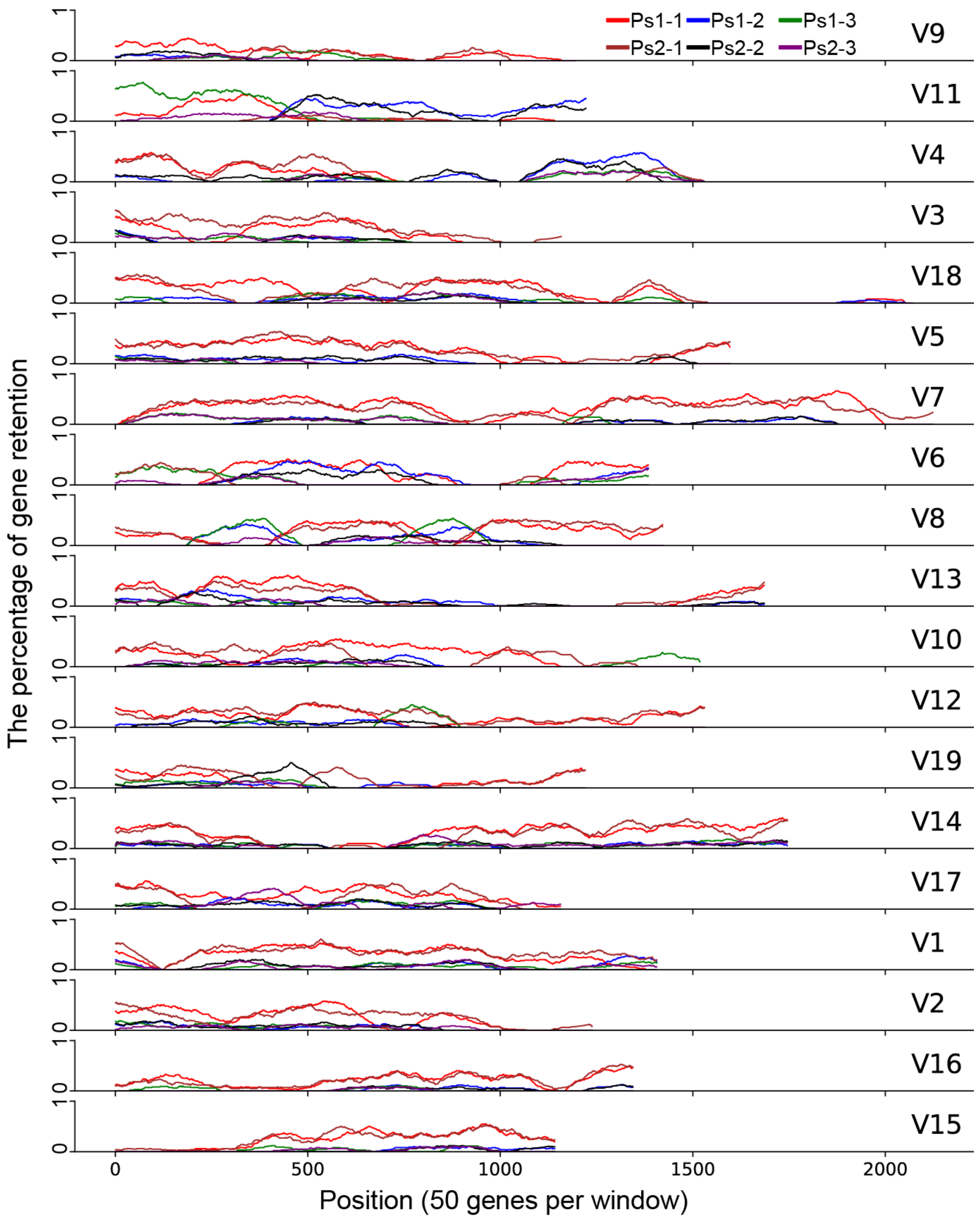

Supplementary Figure 19. Percentages of the retained genes on each putative ancestral core-eudicot chromosome. Names of the colored lines are the same in Figure 2a. X- and y-axis are the number of sliding windows and percentage of gene retention. Source data are provided as a Source Data file.

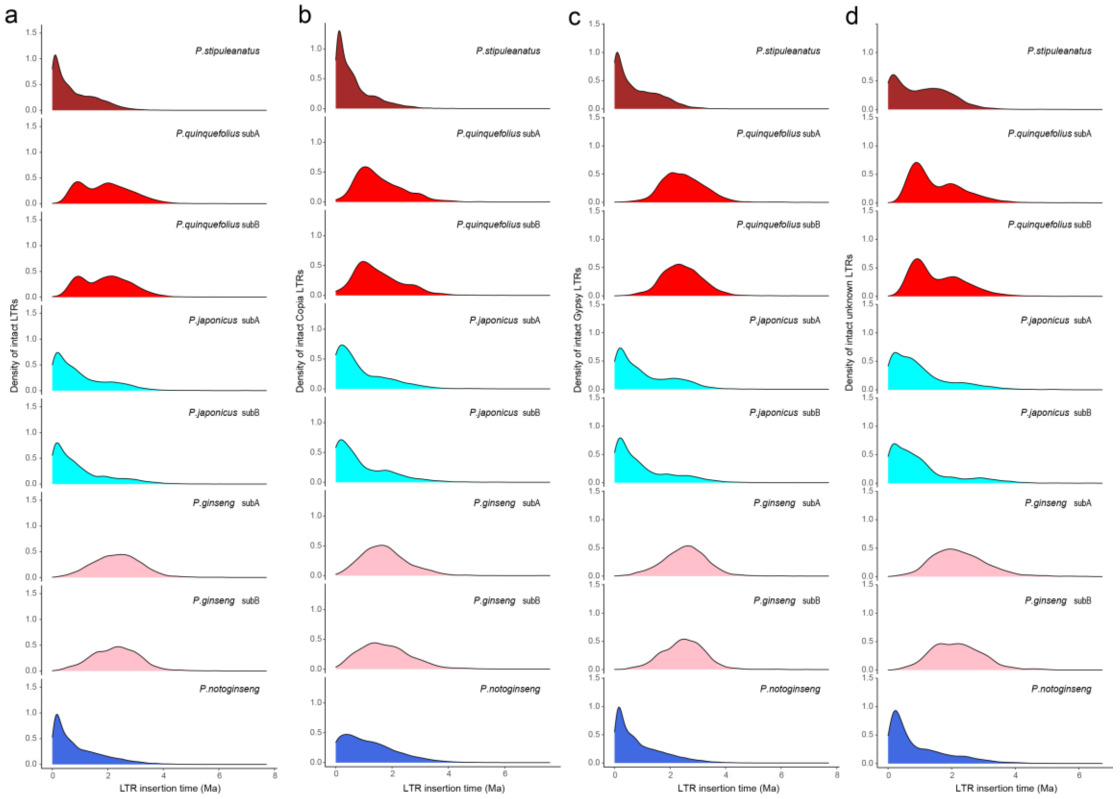

Supplementary Figure 20. Burst patterns of the long terminal repeats (LTRs) in the five *Panax* species. X- and Y-axis are the density of intact LTRs, and LTR insertion time (million year ago (Ma)), respectively. From left to right are all LTRs (a), *Copia* (b), *Gypsy* (c) and unknown LTRs (d). Source data are provided as a Source Data file.

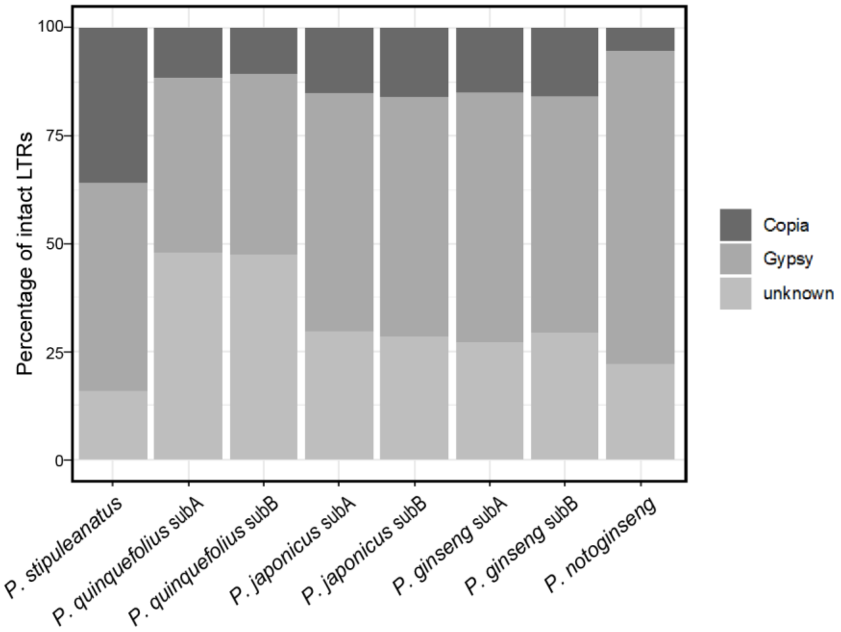

Supplementary Figure 21. Proportions of the three long terminal repeat (LTR) families in the five *Panax* species. Source data are provided as a Source Data file.

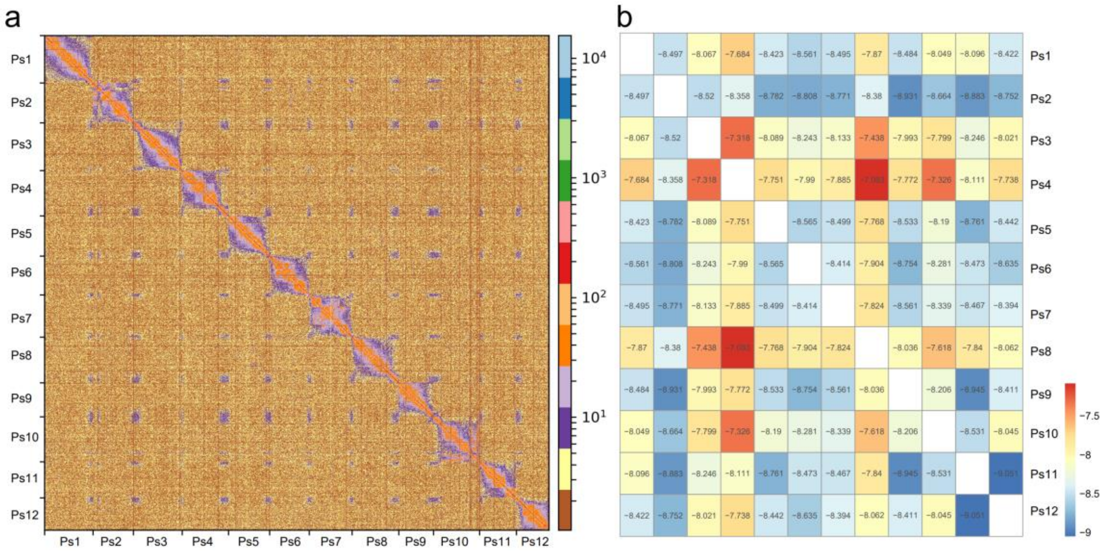

Supplementary Figure 22. Heatmap of chromatin interactions at 100-Kb resolution. (a) Inter- and intra-chromatin interactions for each 100-Kb sliding window. The degree of interactions is shown by the color scheme. (b) Inter-chromatin interactions among the 12 modern chromosomes with all 100-Kb sliding bins combined. Source data are provided as a Source Data file.

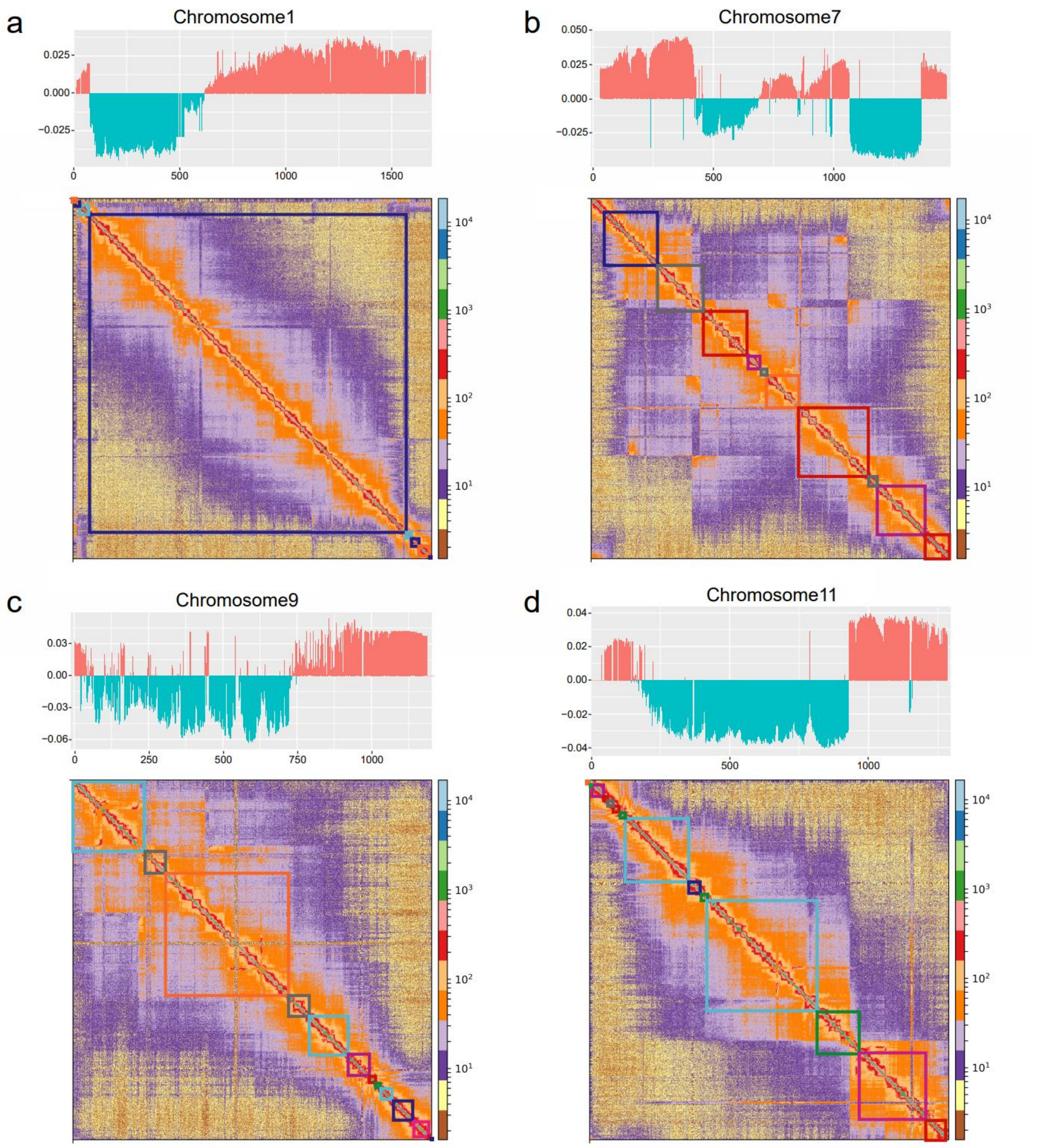

Supplementary Figure 23. Three-dimension (3-D) genome architecture of the modern *Panax* chromosomes. Each chromosome is shown with an independent subpanel. The four subpanels (a-d) represent the modern chromosome 1, chromosome 7, chromosome 9 and chromosome 11, respectively. For each subpanel, on the top, activated (red) and inactivated (blue) compartments at 100-Kb resolution are shown. The X- and Y-axis represent the numbers of 100-Kb sliding bins and PCA eigenvectors of A/B compartments, respectively. At the bottom, heatmaps of the chromatin interactions at 100-Kb resolution are shown. The color scheme on the right indicates the levels of chromatin interaction between the 100-Kb sliding bins. Colored boxes within the heatmap are the ancestral core-eudicot chromosomes. Colors and names of these ancestral chromosomes are the same as in Figure 1. The grey colored box represents the homologous genomic region that was lost in the extant grape genome. Source data are provided as a Source Data file.

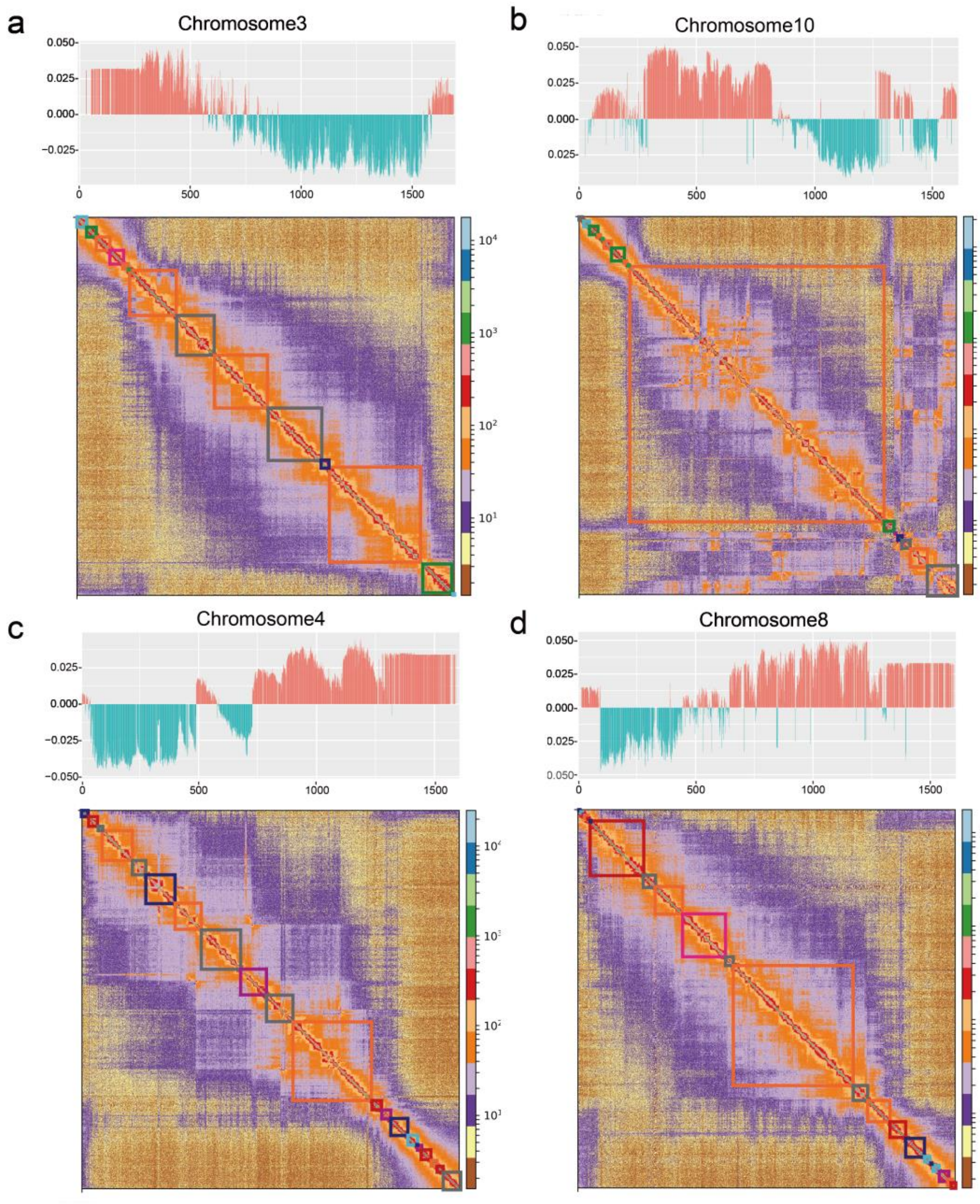

Supplementary Figure 24. Three-dimension (3-D) genome architecture of the modern *Panax* chromosomes. Each chromosome is shown with an independent subpanel. The four subpanels (a-d) represent the modern chromosome 3, chromosome 10, chromosome 4 and chromosome 8, respectively. Interpretation is as in Supplementary Figure 23. Source data are provided as a Source Data file.

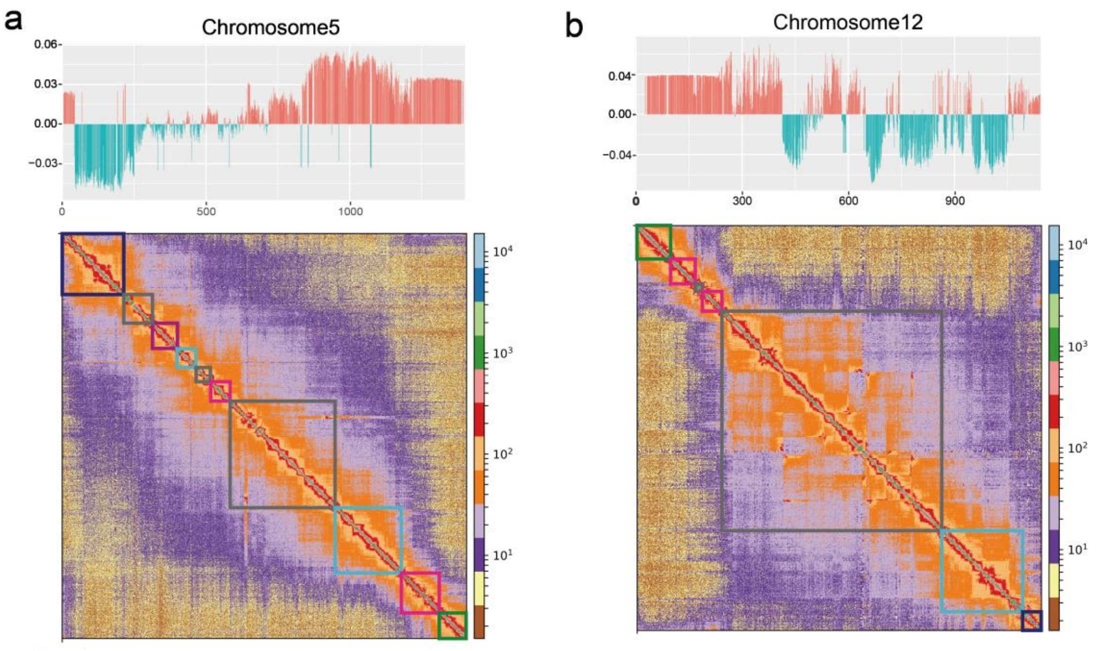

Supplementary Figure 25. Three-dimension (3-D) genome architecture of the modern *Panax* chromosomes. Each chromosome is shown with an independent subpanel. The two subpanels (a-b) represent the modern chromosome 2 and chromosome 12, respectively. Interpretation is as in Supplementary Figure 23. Source data are provided as a Source Data file.

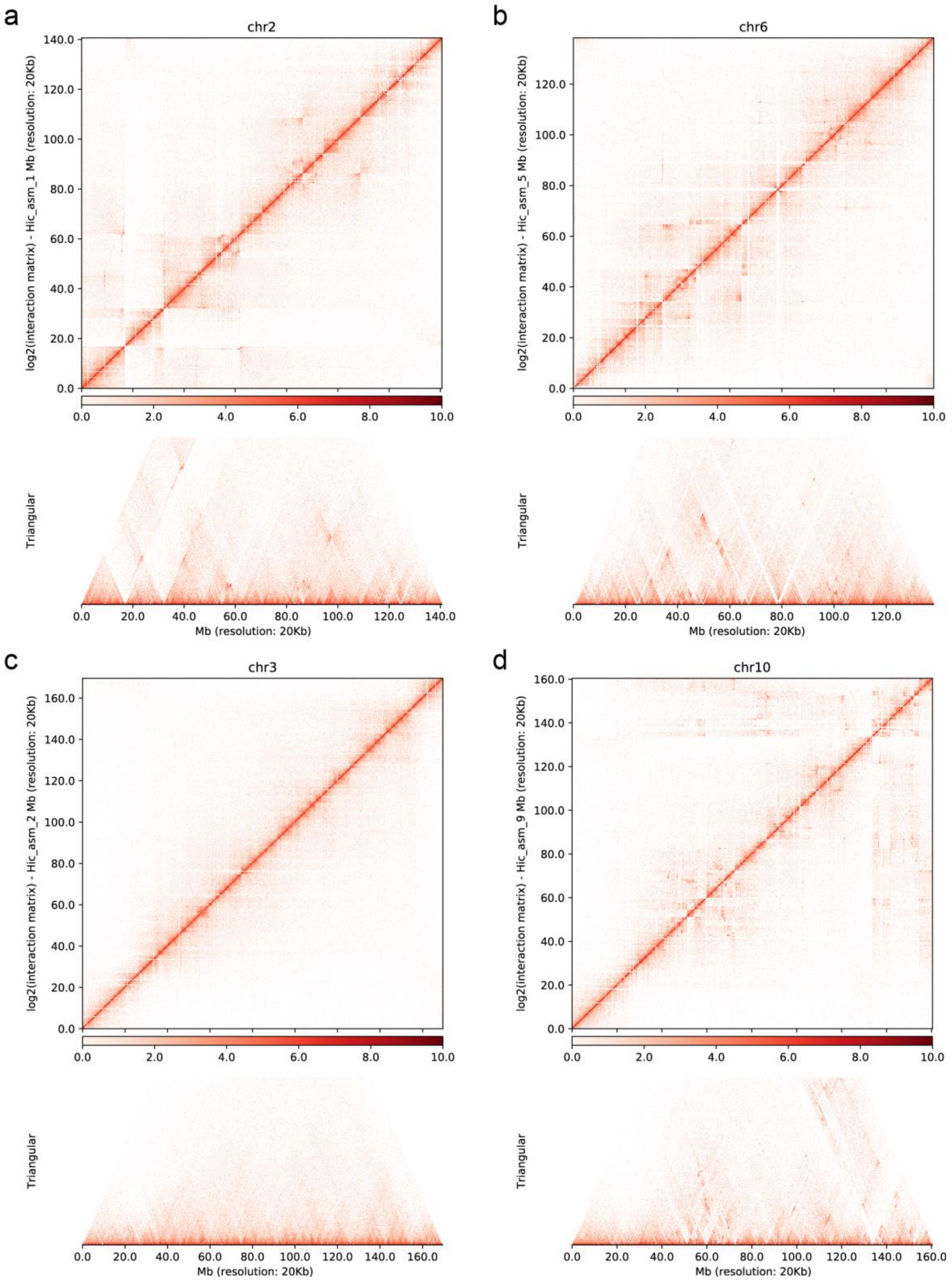

Supplementary Figure 26. Intra-chromosomal interaction map of the *Panax stipuleanatus* based on Hi-C data at 20-Kb resolution. Each chromosome is shown with an independent subpanel. The four subpanels (a-d) present the modern chromosome 2, chromosome 6, chromosome 3 and chromosome 10, respectively. The same interaction matrix of TAD-like structures is also converted to a visualized format in Figure 2c and Figure 3c. Detailed information of the TAD-like structures is given in Supplementary Data 6.

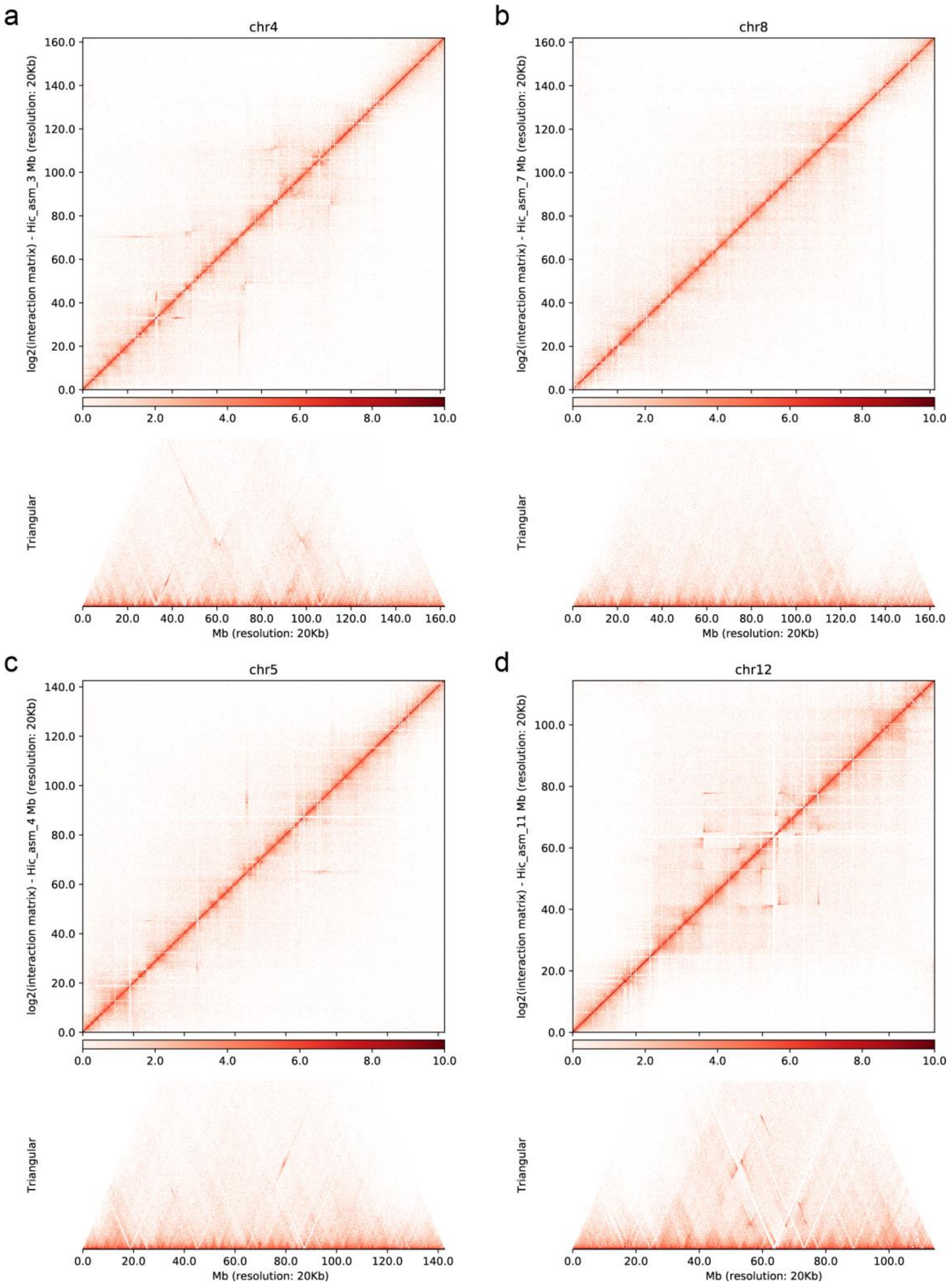

Supplementary Figure 27. Intra-chromosomal interaction map of the *Panax stipuleanatus* based on Hi-C data at 20-Kb resolution. Each chromosome is shown with an independent subpanel. The four subpanels (a-d) present the modern chromosome 4, chromosome 8, chromosome 5 and chromosome 12, respectively. Interpretation is as in Supplementary Figure 26.

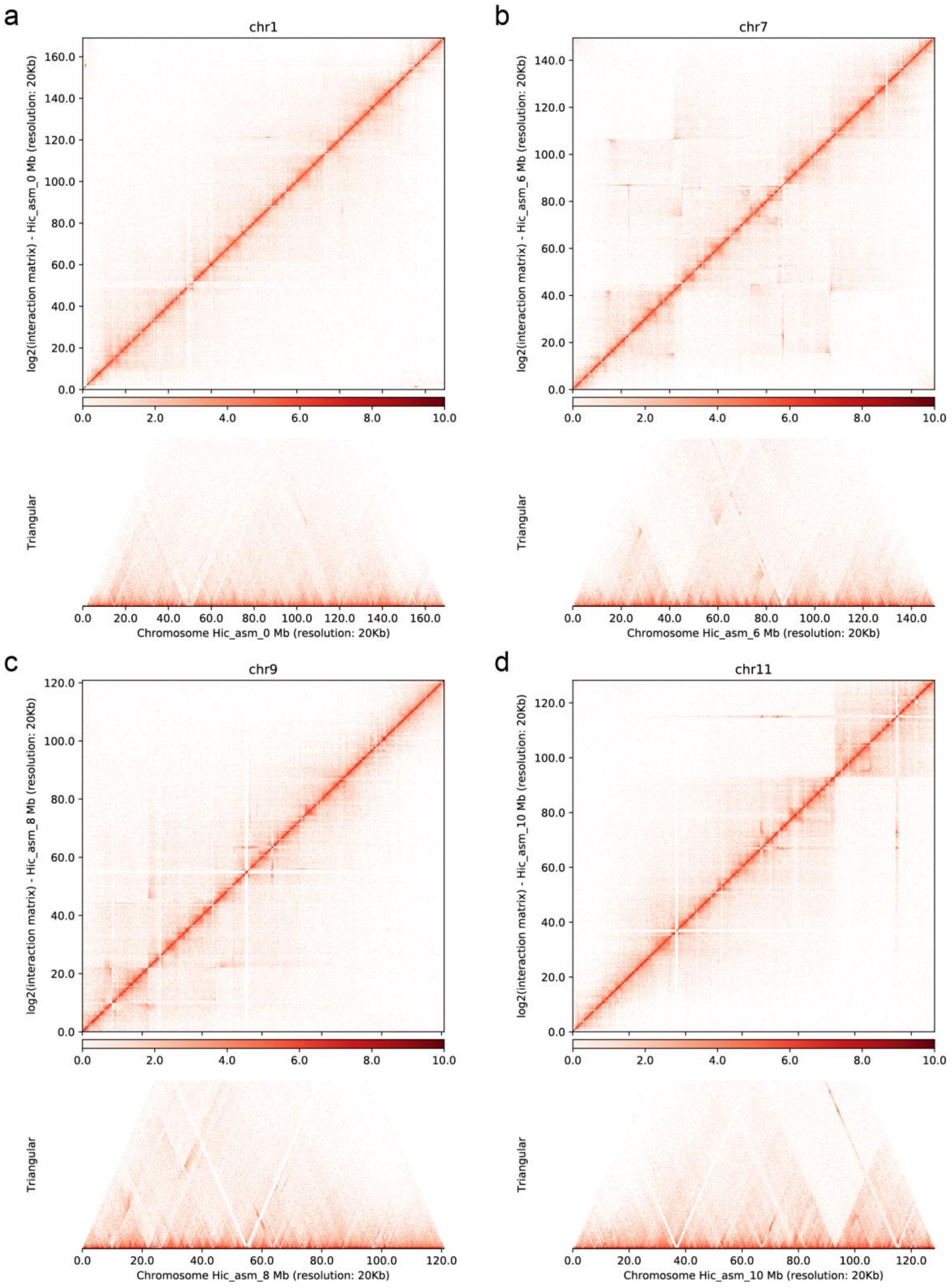

Supplementary Figure 28. Intra-chromosomal interaction map of the *Panax stipuleanatus* based on Hi-C data at 20-Kb resolution. Each chromosome is shown with an independent subpanel. The four subpanels (a-d) present the modern chromosome 1, chromosome 7, chromosome 9 and chromosome 11, respectively. Interpretation is as in Supplementary Figure 26.

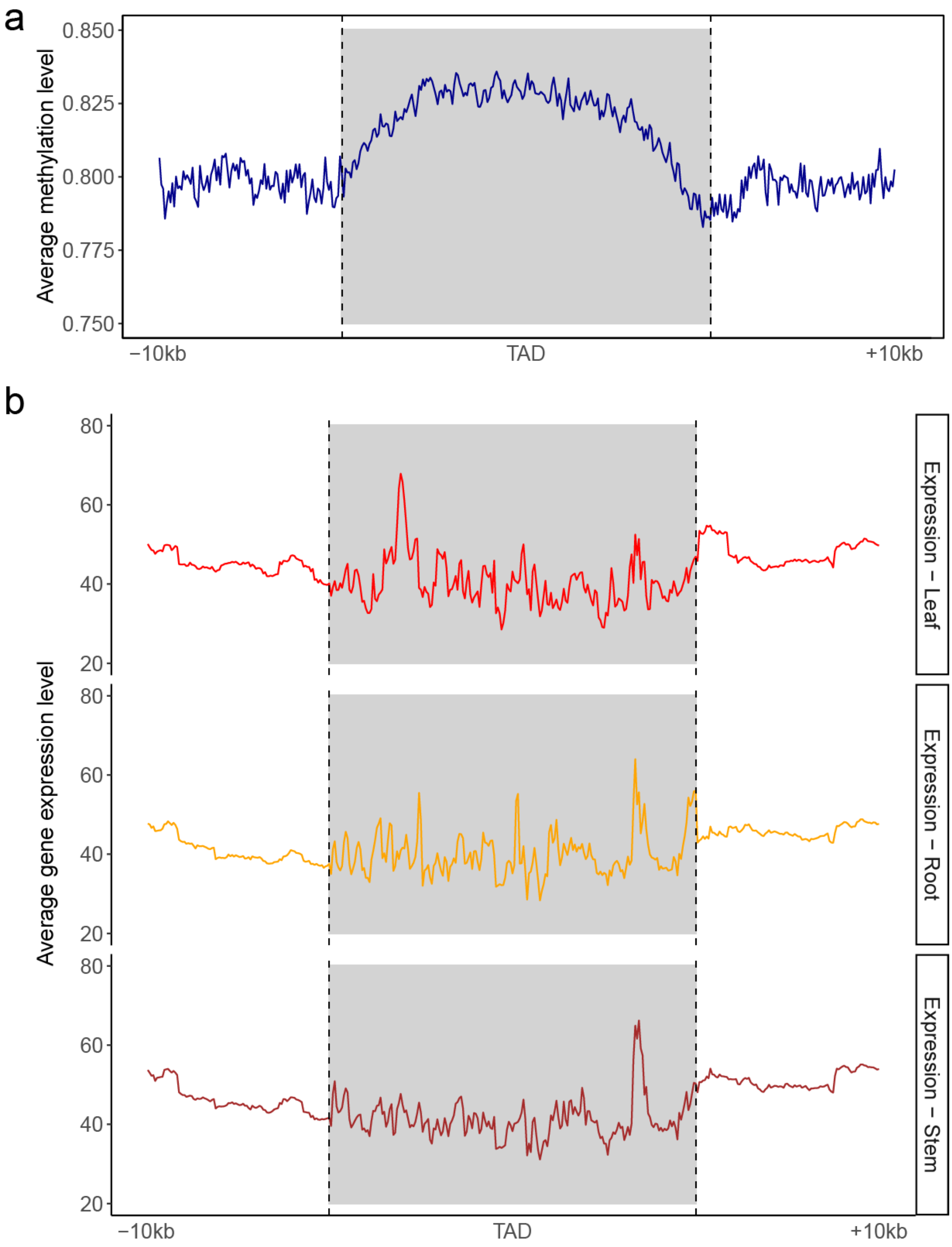

Supplementary Figure 29. Overall patterns of the cytosine methylation (a) and gene expression (b) within the TAD-like structure and its up-/down-stream regions. Source data are provided as a Source Data file.

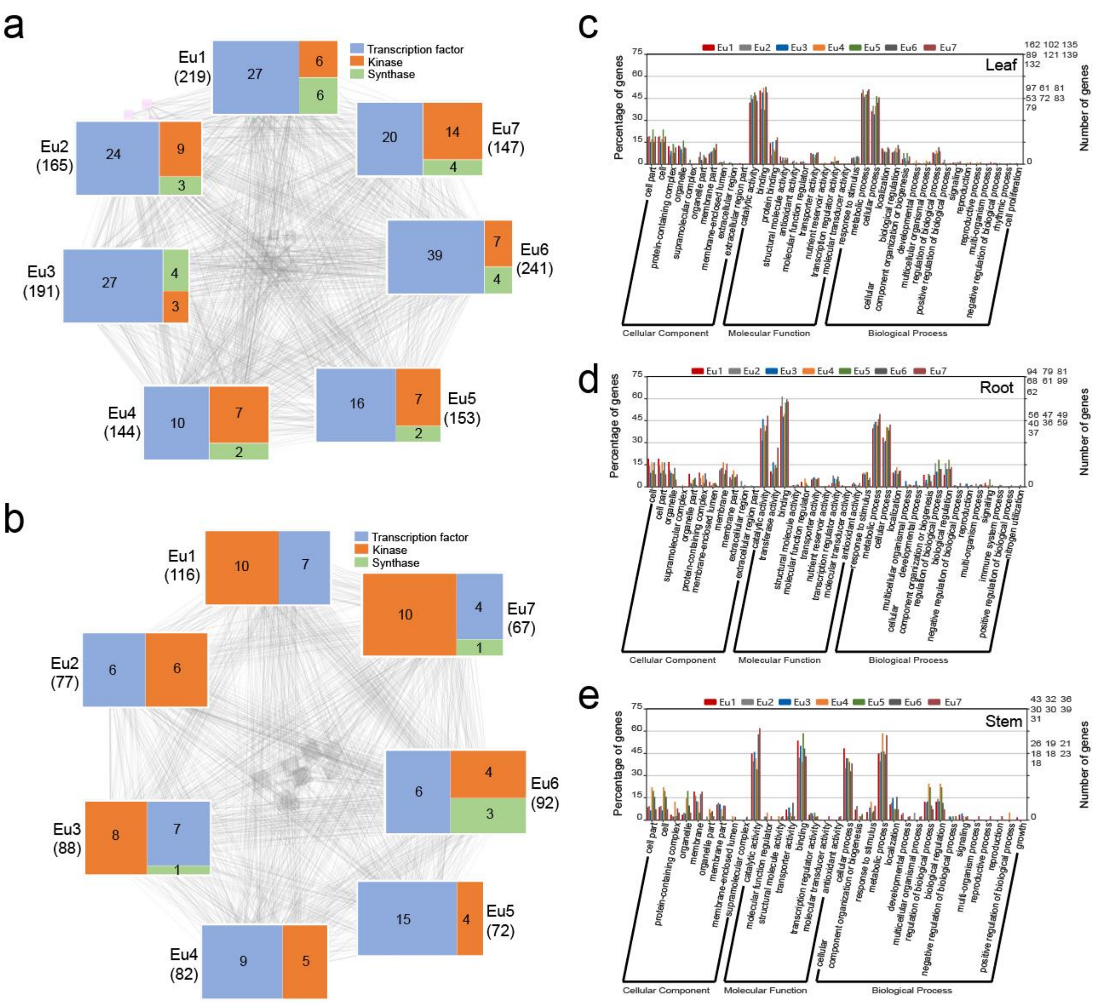

Supplementary Figure 30. Co-expression networks of the ancestral core-eudicot genes involved in the root (a) and stem (b) development in extant *Panax* species. Eight gene clusters were defined according to their originations. Eu1-Eu7 are the seven ancestral eudicot ancestral-chromosomes. Numbers below each ancestral core-eudicot chromosome are the total number of identified ancestral genes. Grey dots in the middle are the unassigned ancestral genes. Colors and numbers represent the functions and numbers of ancestral genes involved in the leaf development. Grey lines indicate the ancestral gene interactions. (c-e) Go term analyses of the ancestral genes identified in the most correlated regulatory modules in leaf, root and stem tissues. The seven types of colors represent the seven ancestral core-eudicot chromosomes. Source data are provided as a Source Data file.

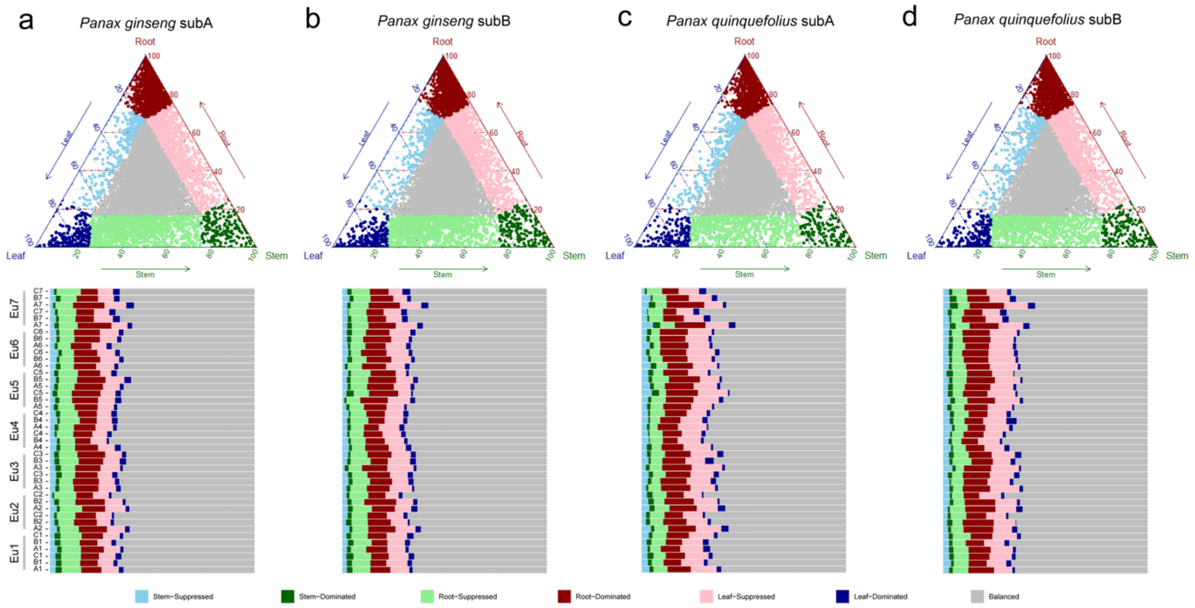

Supplementary Figure 31. Expression patterns of the ancestral core-eudicot genes in the extant *Panax ginseng* (a-b) and *Panax quinquefolius* (c-d) genomes. On top, the ternary plot indicates the expression patterns of retained ancestral genes in modern genomes. Each circle is a triad showing relative expression abundance of each ancestral gene in the leaf, root and stem tissues. Triads in vertices correspond to the three tissue-dominant expression categories, whereas triads close to edges and between vertices correspond to the three tissue-suppressed expression categories. Balanced triads are shown in gray. At bottom, the percentage of triads in each category of the ancestral genes is shown. The 42 bars from top to bottom are the duplicated ancestral core-eudicot chromosomes (Eu1-Eu7). Each ancestral chromosome contains six homologous genomic regions in extant genomes. Names of each ancestral chromosome are the same as in Figure 1. Source data are provided as a Source Data file.

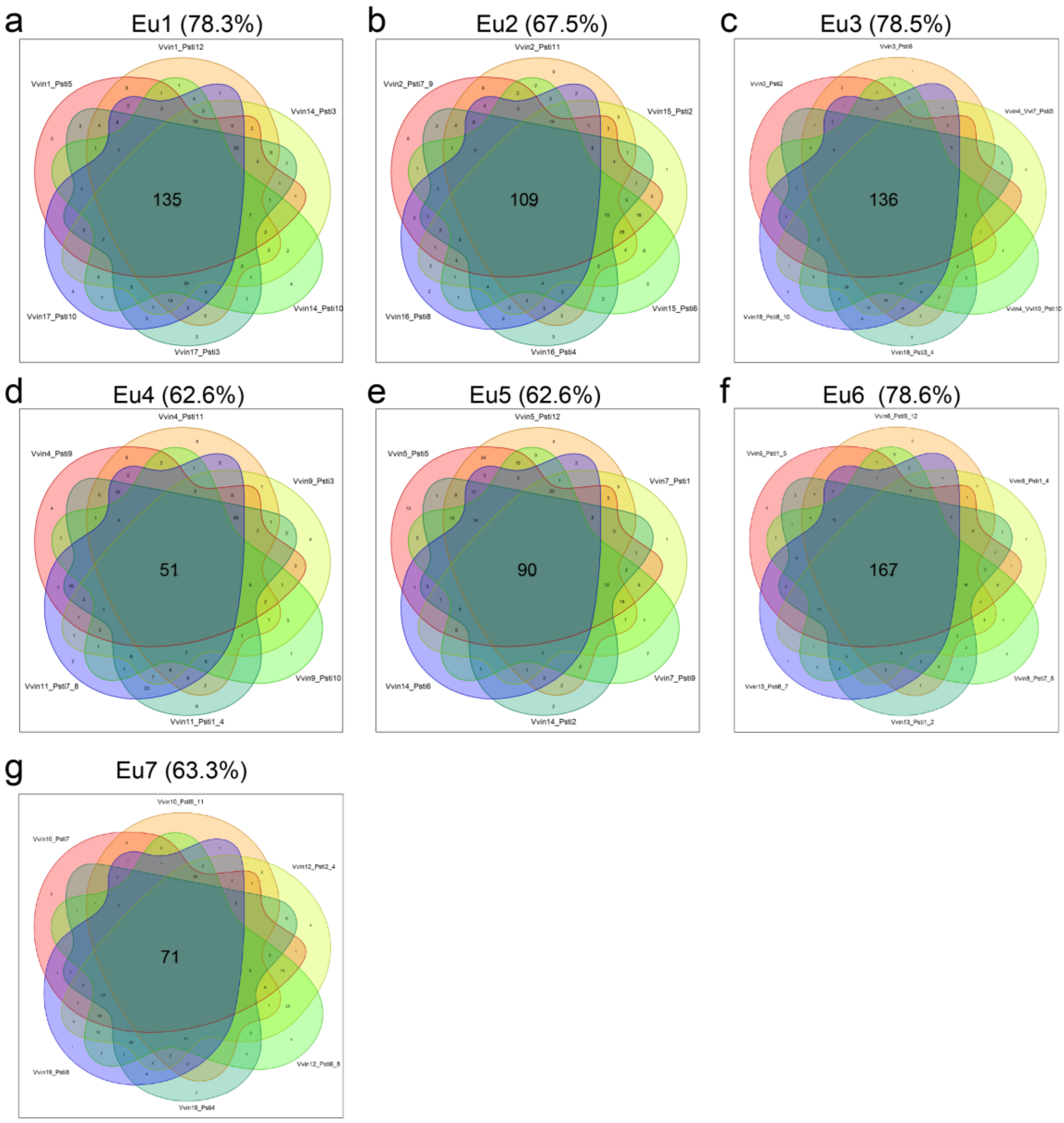

Supplementary Figure 32. Venn diagram of the KEGG pathways of the seven ancestral core-eudicot chromosomes. The seven subpanels (a-g) represent the seven ancestral core-eudicot chromosomes (Eu1-Eu7). Each ancestral core-eudicot chromosome consists of six homologous genomic regions that were derived from the same ancestral core-eudicot chromosome. Numbers in the Venn diagram are KEGG categories identified on each duplicated ancestral core-eudicot chromosome. Source data are provided as a Source Data file.

Expression type	Leaf dominated							Leaf suppressed						
Eudicot ID	Eu1	Eu2	Eu3	Eu4	Eu5	Eu6	Eu7	Eu1	Eu2	Eu3	Eu4	Eu5	Eu6	Eu7
1	77.14%	86.09%	53.95%	82.93%	67.82%	63.16%	69.16%	53.53%	60.14%	29.88%	72.92%	65.45%	55.02%	48.33%
2	20.00%	11.26%	26.32%	12.20%	24.14%	22.56%	22.43%	18.82%	31.47%	34.02%	18.75%	22.73%	26.20%	26.32%
3	2.86%	1.99%	17.11%	4.88%	6.90%	12.78%	6.54%	15.29%	6.99%	19.50%	7.64%	10.00%	8.30%	19.62%
4	0.00%	0.66%	1.32%	0.00%	1.15%	1.50%	0.93%	8.82%	1.40%	11.20%	0.69%	1.82%	6.99%	4.31%
5	0.00%	0.00%	1.32%	0.00%	0.00%	0.00%	0.93%	2.35%	0.00%	5.39%	0.00%	0.00%	3.06%	1.44%
6	0.00%	0.00%	0.00%	0.00%	0.00%	0.00%	0.00%	1.18%	0.00%	0.00%	0.00%	0.00%	0.44%	0.00%
>half	0.00%	0.66%	2.63%	0.00%	1.15%	1.50%	1.87%	12.35%	1.40%	16.60%	0.69%	1.82%	10.48%	5.74%
Expression type	Root dominated							Root suppressed						
Eudicot ID	Eu1	Eu2	Eu3	Eu4	Eu5	Eu6	Eu7	Eu1	Eu2	Eu3	Eu4	Eu5	Eu6	Eu7
1	54.76%	68.37%	62.50%	79.34%	70.27%	53.94%	53.00%	61.04%	77.44%	59.91%	71.35%	62.33%	40.44%	50.38%
2	27.78%	26.53%	25.89%	16.53%	22.30%	24.24%	38.00%	22.73%	15.79%	29.28%	23.96%	28.08%	36.44%	33.83%
3	11.90%	3.06%	9.82%	3.31%	5.41%	12.73%	8.00%	11.04%	5.26%	8.11%	3.65%	8.90%	19.11%	9.77%
4	3.17%	2.04%	1.79%	0.83%	1.35%	5.45%	1.00%	3.90%	1.50%	1.35%	1.04%	0.68%	3.56%	4.51%
5	1.59%	0.00%	0.00%	0.00%	0.68%	3.03%	0.00%	0.65%	0.00%	1.35%	0.00%	0.00%	0.44%	1.50%
6	0.79%	0.00%	0.00%	0.00%	0.00%	0.61%	0.00%	0.65%	0.00%	0.00%	0.00%	0.00%	0.00%	0.00%
>half	5.56%	2.04%	1.79%	0.83%	2.03%	9.09%	1.00%	5.19%	1.50%	2.70%	1.04%	0.68%	4.00%	6.02%
Expression type	Stem dominated							Stem suppressed						
Eudicot ID	Eu1	Eu2	Eu3	Eu4	Eu5	Eu6	Eu7	Eu1	Eu2	Eu3	Eu4	Eu5	Eu6	Eu7
1	63.77%	90.74%	73.26%	84.91%	77.50%	68.22%	61.29%	58.12%	89.83%	70.49%	91.30%	79.07%	80.82%	86.79%
2	27.54%	7.41%	20.93%	15.09%	17.50%	26.17%	33.06%	36.75%	8.47%	27.87%	6.52%	18.60%	13.70%	13.21%
3	4.35%	1.85%	4.65%	0.00%	5.00%	4.67%	4.84%	5.13%	1.69%	1.64%	2.17%	2.33%	4.11%	0.00%
4	4.35%	0.00%	1.16%	0.00%	0.00%	0.93%	0.81%	0.00%	0.00%	0.00%	0.00%	0.00%	1.37%	0.00%
5	0.00%	0.00%	0.00%	0.00%	0.00%	0.00%	0.00%	0.00%	0.00%	0.00%	0.00%	0.00%	0.00%	0.00%
6	0.00%	0.00%	0.00%	0.00%	0.00%	0.00%	0.00%	0.00%	0.00%	0.00%	0.00%	0.00%	0.00%	0.00%
>half	4.35%	0.00%	1.16%	0.00%	0.00%	0.93%	0.81%	0.00%	0.00%	0.00%	0.00%	0.00%	1.37%	0.00%
Expression type	Balanced expression													
Eudicot ID	Eu1	Eu2	Eu3	Eu4	Eu5	Eu6	Eu7							
1	8.92%	17.26%	8.36%	18.59%	17.04%	7.67%	20.40%							
2	12.42%	18.24%	17.34%	19.23%	22.19%	14.70%	25.08%							
3	16.88%	16.61%	13.62%	15.38%	18.65%	11.50%	17.06%							
4	14.01%	16.61%	21.36%	22.76%	16.08%	17.25%	17.73%							
5	21.66%	16.94%	17.65%	16.99%	14.15%	21.09%	14.38%							
6	26.11%	14.33%	21.67%	7.05%	11.90%	27.80%	5.35%							
>half	61.78%	47.88%	60.68%	46.79%	42.12%	66.13%	37.46%							

Supplementary Figure 33. Statistics of the shared and specific KEGG categories among the six ancestral core-eudicot chromosome (Eu1-Eu7) duplicates in the seven gene expression types. The seven gene expression types are defined as the same as Figure 4. Eudicot ID represents the seven ancestral core-eudicot chromosomes (see Figure 1). In the leaf column, numbers from 1 to 6 indicate the specific (=1) and shared (2-6) KEGG categories among the six ancestral chromosome duplicates. Numbers below the seven ancestral chromosomes (Eu1-Eu7) show the percentage of specific and shared KEGG categories. ">half" indicates the KEGG categories that are shared among at least four of the six ancestral-chromosomes duplicates. The Balanced expression type (37.46-66.13%) showed obviously higher proportions of ">half" KEGG categories compared to the other six expression types (0.00-10.48%). Source data are provided as a Source Data file.

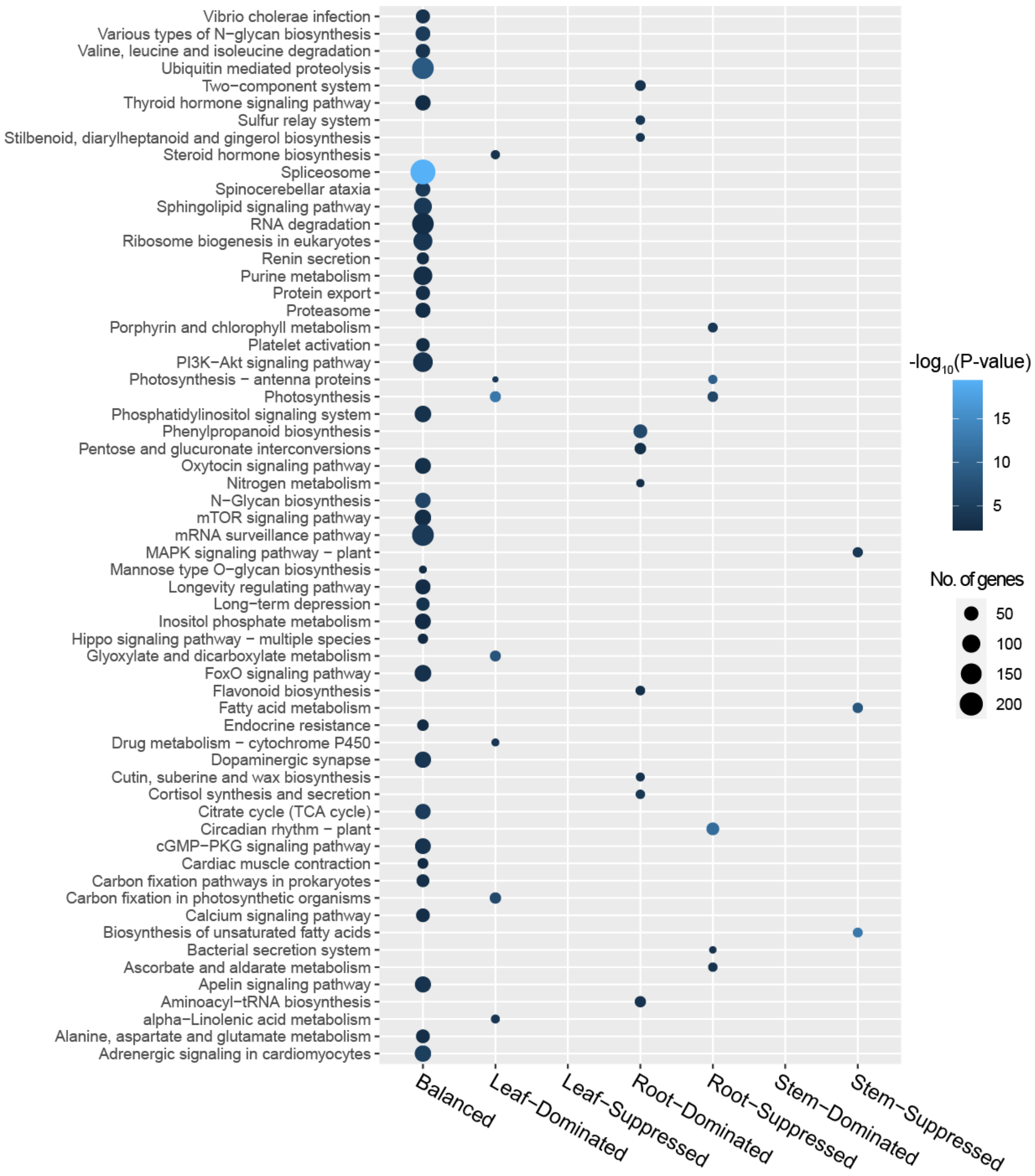

Supplementary Figure 34. KEGG enrichment of the seven gene-expression types in extant ginseng genomes. The seven expression types are the same as in Figure 4. Source data are provided as a Source Data file.

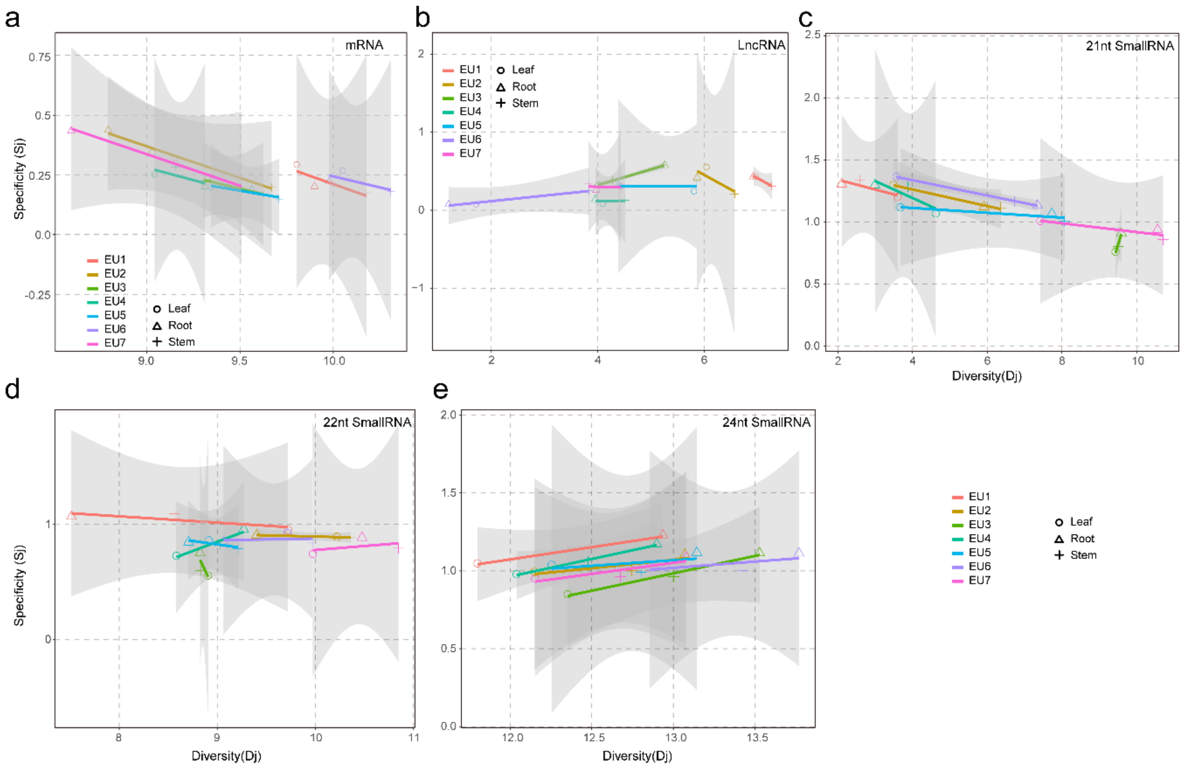

Supplementary Figure 35. Expression dynamics of protein-coding genes (a) and non-coding (b-e) RNAs. The X- and Y-axis are the expression diversity (Dj) and specificity (Sj) of the ancestral core-eudicot genes, respectively. The circle, triangle and cross symbols represent leaf, root and stem tissues, respectively. Colored lines represent the seven ancestral core-eudicot chromosomes. Differences in Dj and Sj values indicate high expression diversity among the ancestral chromosomes and high expression specificity among the three tissues. The edges of each shaded area are the error bands. Source data are provided as a Source Data file.

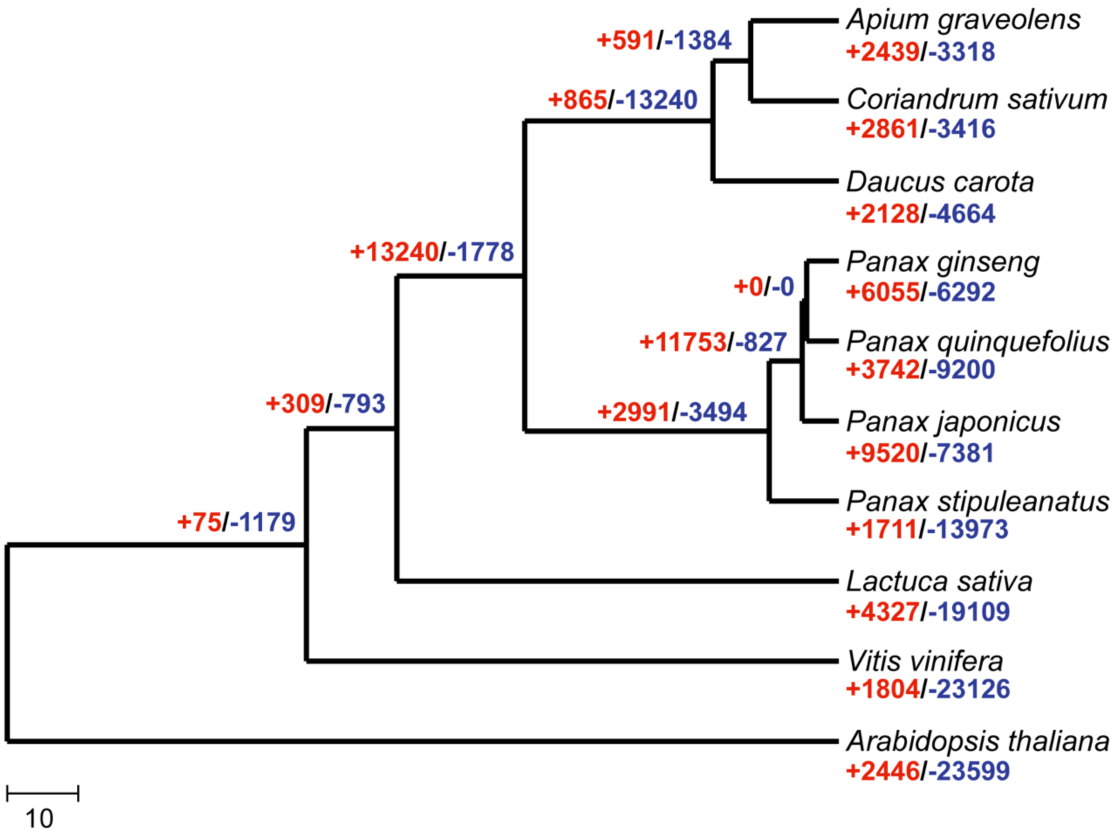

Supplementary Figure 36. Contraction and expansion history of the gene families in the selected core-eudicot species. Red and blue numbers on the branches and under each species name are the expansion and contraction gene families. Source data are provided as a Source Data file.

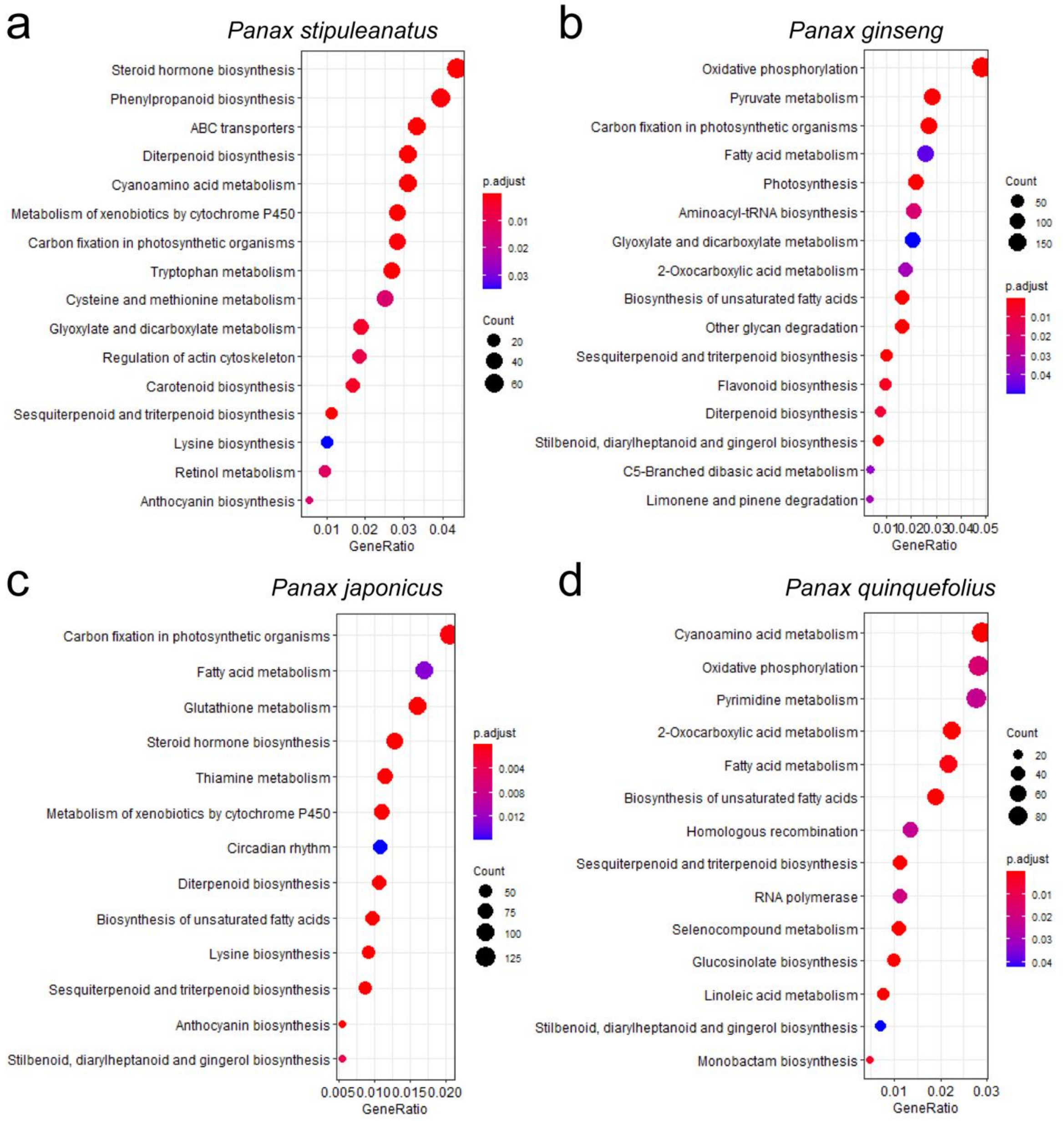

Supplementary Figure 37. KEGG enrichment of the expanded gene families in the selected core-eudicot species. The four subpanels (a-d) represent enriched KEGG terms in the *Panax stipuleanatus*, *Panax ginseng*, *Panax japonicus* and *Panax quinquefolius*, respectively. Source data are provided as a Source Data file.

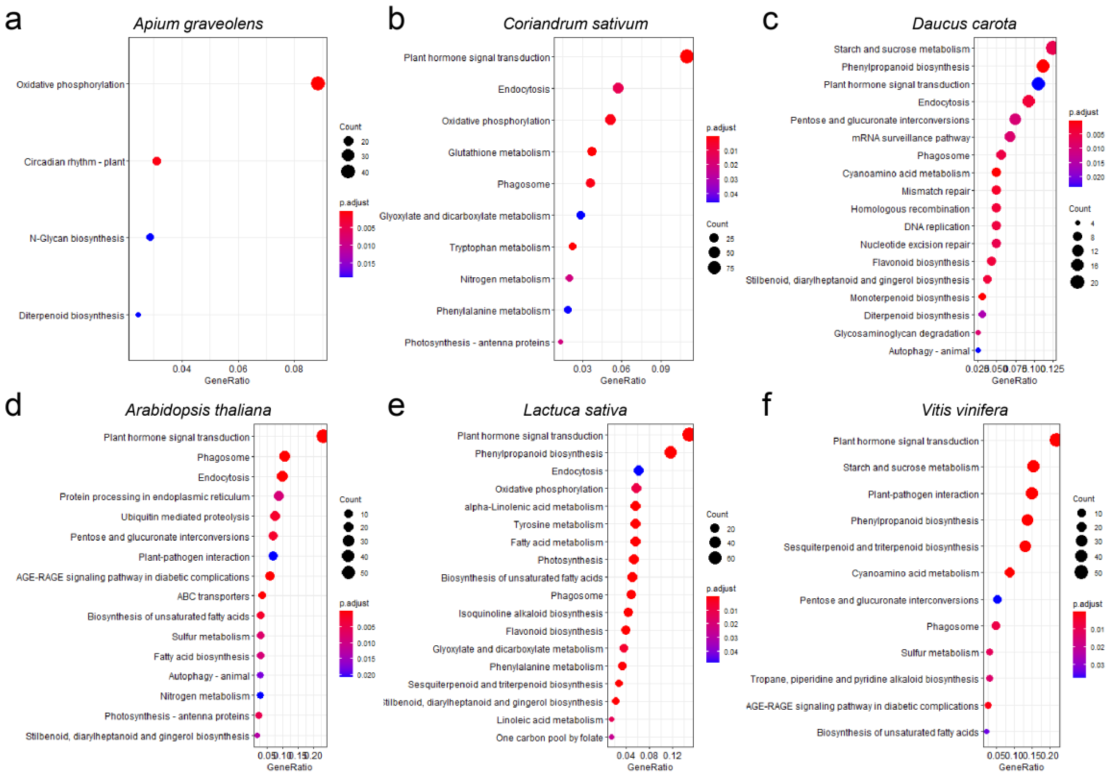

Supplementary Figure 38. KEGG enrichment of the expanded gene families in the selected core-eudicot species. The first two subpanels (a-c) represent enriched KEGG terms in the three Apiaceae species (*Apium graveolens*, *Coriandrum sativum* and *Daucus carota*). The last four subpanels (d-f) represent enriched KEGG terms in the other three genetically distant species (*Arabidopsis thaliana*, *Lactuca sativa* and *Vitis vinifera*).Source data are provided as a Source Data file.

a

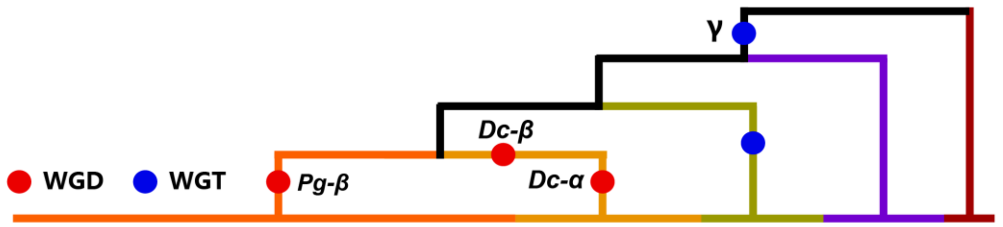

	PGsubA	PGsubB	PJsubA	PJsubB	PQsubA	PQsubB	PN	PS	DC	AG	CS	LS	PGR	VV	AT	AC
Total	173	175	145	156	159	146	194	180	200	156	250	329	240	207	244	437
51-clan	2	2	2	2	2	1	3	2	1	1	0	1	4	2	1	2
71-clan	81	83	72	60	83	72	107	85	116	58	117	213	117	94	155	250
72-clan	25	24	17	23	22	23	23	30	21	28	45	27	38	44	19	64
74-clan	2	3	4	3	3	4	2	2	2	1	5	8	5	4	2	5
86-clan	23	24	17	25	18	17	25	22	24	34	32	41	22	20	33	31
97-clan	3	3	4	4	2	2	3	3	3	3	2	3	3	3	3	5
710-clan	1	1	2	1	1	0	1	1	1	1	1	1	1	0	4	3
711-clan	2	2	2	2	0	2	1	1	1	1	9	1	1	1	1	1
85-clan	34	33	25	36	28	25	29	34	31	29	39	34	49	39	26	76

b

CYP85	2	3	3	3	2	2	3	3	3	3	5	1	2	3	2	3
CYP87	3	4	2	2	3	4	2	4	6	5	5	2	2	8	1	13
CYP88	6	3	2	4	5	2	3	3	2	4	3	1	6	2	2	5
CYP90	6	6	6	7	6	4	6	6	4	3	3	7	6	4	4	18
CYP702	0	0	0	0	0	0	0	0	0	0	0	0	0	0	2	0
CYP707	6	6	2	5	4	5	4	5	7	8	12	6	4	5	4	15
CYP708	0	0	0	0	0	0	0	0	0	0	0	0	0	0	4	0
CYP718	1	1	0	3	1	1	1	1	1	0	1	2	1	1	1	1
CYP720	1	1	1	2	1	1	0	1	1	1	1	1	1	1	1	1
CYP722	0	0	1	0	0	0	0	1	0	0	0	1	0	2	1	1
CYP724	2	2	2	2	1	3	2	2	3	1	3	1	2	2	1	2
CYP716	7	7	6	8	5	3	8	8	4	4	6	12	25	11	3	17

Supplementary Figure 39. Polyploidization history and identification of CYP450 in the ginseng genus and the other eight selected core-eudicot species. (a) Gene numbers of the nine major CYP450 clades in each selected species. Red and blue solid circles are whole genome duplication and triplications. (b) Gene numbers of the 12 subfamilies in the CYP85 clade. Colors from blue to red indicate the increasing copy numbers. PG, *Panax ginseng*; PJ, *Panax japonicus*; PQ, *Panax quinquefolius*; PN, *Panax notoginseng*; PS, *Panax stipuleanatus*; DC, *Daucus carota*; AG, *Apium graveolens*; CS, *Coriandrum sativum*; LS, *Lactuca sativa*; PGR, *Platycodon grandifloras*; VV, *Vitis vinifera*; AT, *Arabidopsis thaliana*; AC, *Aquilegia viridiflora*. Source data are provided as a Source Data file.

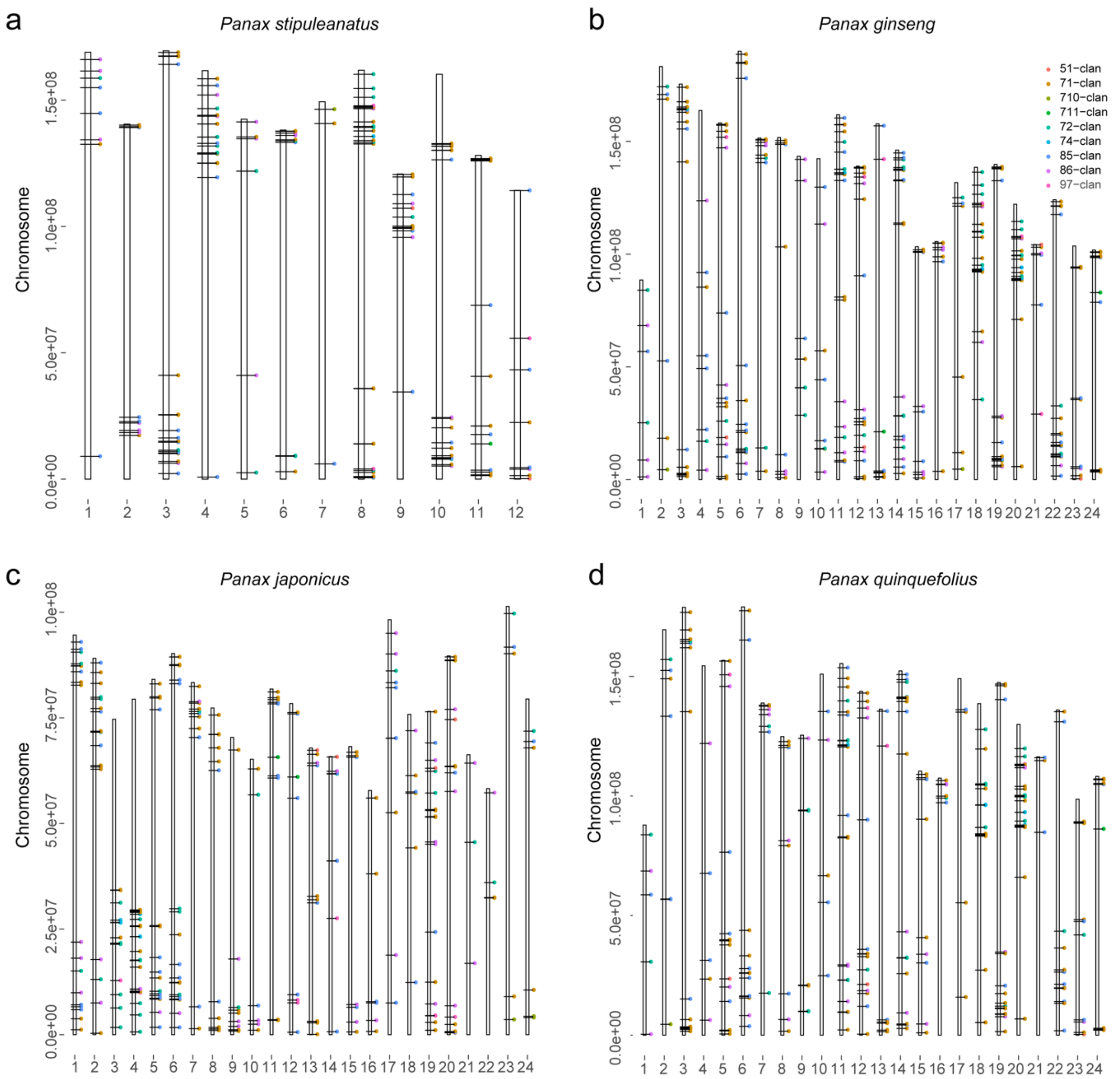

Supplementary Figure 40. Distribution of the CYP450 superfamily in the four *Panax* species. The four subpanels (a-d) represent *Panax stipuleanatus*, *Panax ginseng*, *Panax japonicus* and *Panax quinquefolius*, respectively. Colored dots are the nine major clades identified in Arabidopsis. Black line indicates the physical position of these CYP450 genes on the chromosomes of the four *Panax* species. Source data are provided as a Source Data file.

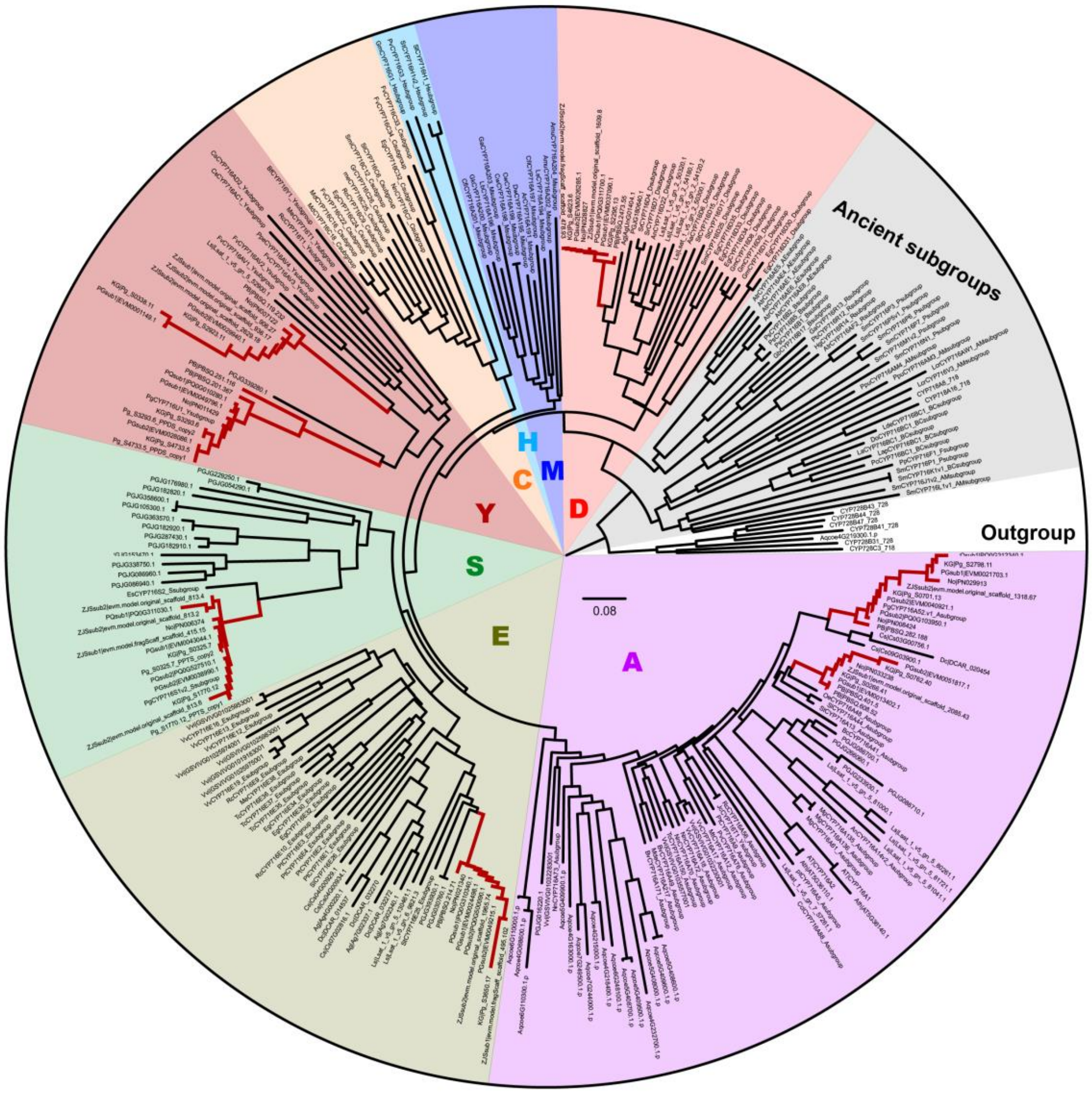

Supplementary Figure 41. Neighbor-Joining (NJ) tree of the CYP716 subfamily in the selected core-eudicot species. Branches marked in red color are ginseng species genomes generated from this study. All the other data were obtained from previous study[43]. The characters (A, C, D, E, M, S and Y) are subgroups of the CYP716 subfamily. Source data are provided as a Source Data file.

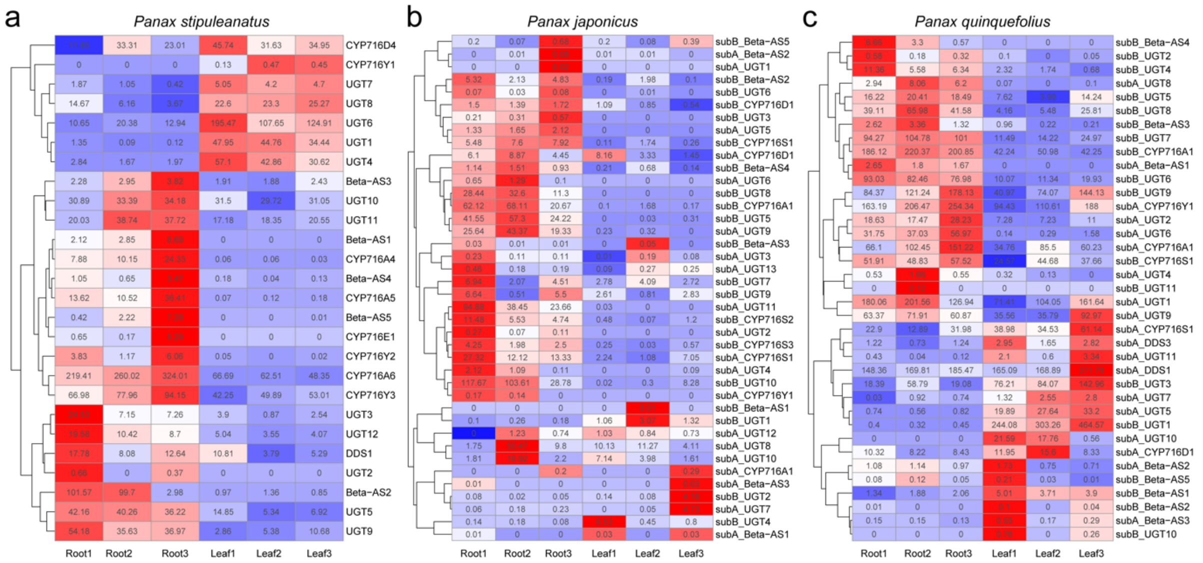

Supplementary Figure 42. Heatmap of the expression patterns of the ginsenoside biosynthesis genes in *Panax stipuleanatus* (a), *Panax japonicus* (b) and *Panax quinquefolius* (c), respectively. Source data are provided as a Source Data file.

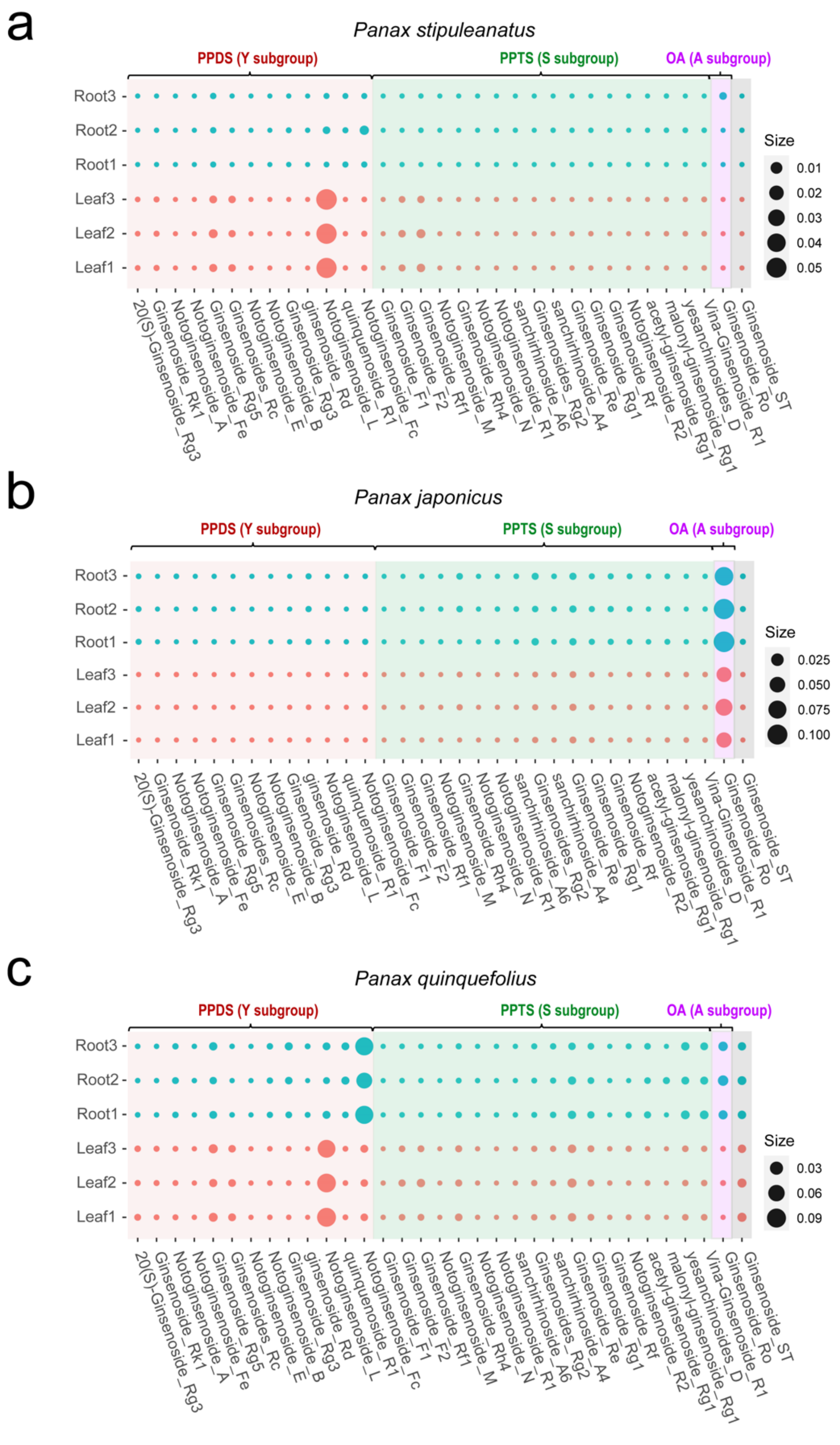

Supplementary Figure 43. Metabolic analyses of the triterpenoid biosynthesis in ginseng root and leaf tissues of the *Panax stipuleanatus* (a), *Panax japonicus* (b) and *Panax quinquefolius* (c), respectively. Name of each ginsenoside is shown at the bottom. Red, green and purple colors indicate the two dammarane and one oleanane type ginsenosides, respectively. Source data are provided as a Source Data file.

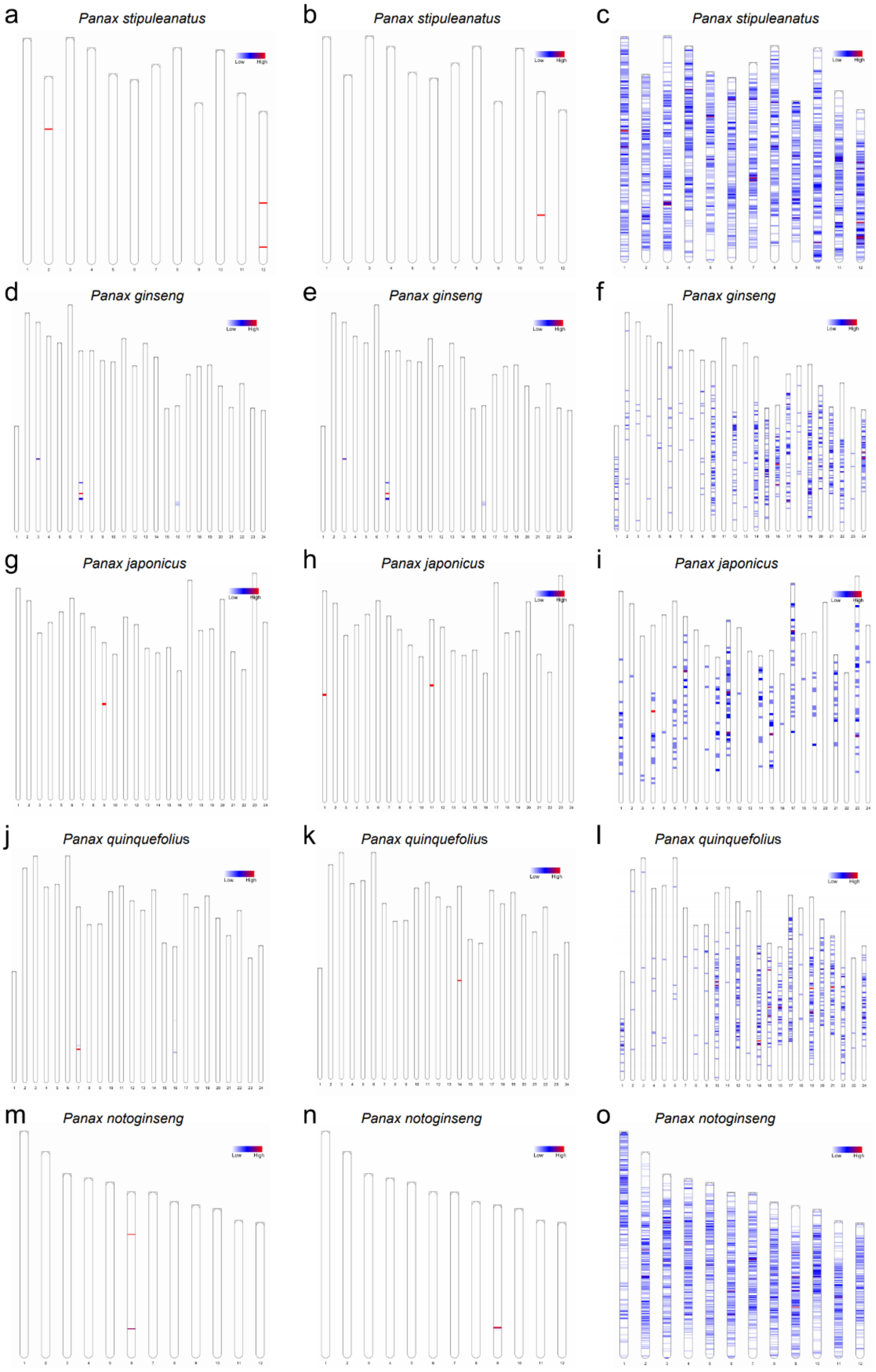

Supplementary Figure 44. Simulated karyotypes of the five *Panax* species based on the fluorescent in situ hybridization (FISH) probes (5S, 45S and *PgDel2*) developed by previous studies[21-23]. (a-c) Karyotypes of *Panax stipuleanatus* simulated with the probes 5S, 45S and PgDel2, respectively. (d-f) Karyotypes of *Panax ginseng* simulated with the probes 5S, 45S and PgDel2, respectively. (g-i)

Karyotypes of *Panax japonicus* simulated with the probes 5S, 45S and PgDel2, respectively. (j-l) Karyotypes of *Panax quinquefolius* simulated with the probes 5S, 45S and PgDel2, respectively. (m-o) Karyotypes of *Panax notoginseng* simulated with the probes 5S, 45S and PgDel2, respectively. Source data are provided as a Source Data file.

Supplementary Table 1. Identification of the subgenomes in the three tetraploid *Panax* species.

Orthologous chromosome	*Panax stipuleanatus*	*Panax ginseng*		*Panax quiquefolium*		*Panax japonicus*		*Panax notoginseng*[#]
		Subgenome A	Subgenome B	Subgenome A	Subgenome B	Subgenome A	Subgenome B	
Chr1*	1	4	10	4	10	18	17	6
Chr2	2	8	15	8	15	16	15	12
Chr3	3	6	22	6	22	5	6	4
Chr4	4	11	14	11	14	2	1	1
Chr5	5	9	1	9	1	22	21	8
Chr6	6	7	16	7	16	9	10	7
Chr7	7	2	17	2	17	24	23	3
Chr8	8	18	20	18	20	3	4	9
Chr9	9	5	12	5	12	20	19	5
Chr10	10	3	19	3	19	8	7	2
Chr11	11	13	24	13	24	12	11	10
Chr12	12	23	21	23	21	13	14	11

*, ID of the orthologous chromosomes named according to *Panax stipuleanatus*. The other numbers in the table are the chromosome ID of the four *Panax* species; #, these results were estimated based on the genome retrieved from previous study[19].

Supplementary Table 2. Number of collinear genes (ancestral core-eudicot genes) between grape and the other selected eudicot species.

Inter-specific comparison	Number of homologous genomic blocks	Number of collinear genes
Lactuca sativa and *Vitis vinifera*	1,863	14,087
Daucus carota and *Vitis vinifera*	1,546	13,985
Panax stipuleanatus and *Vitis vinifera*	1,294	16,010
Panax japonicus and *Vitis vinifera*	2,471	31,729
Panax ginseng and *Vitis vinifera*	2,231	30,042
Panax quiquefolium and *Vitis vinifera*	2,023	25,547

Supplementary Table 3. Pangenomic analyses of the gene families in the four *Panax* species.

Species/subgenome	Species-specific gene family	Dispensable gene family*					Core gene family
		2	3	4	5	6	
Panax ginseng subgenome A	161	1,678	1,337	1,504	2,218	5,562	9,927
Panax ginseng subgenome B	51	1,074	1,114	1,374	2,159	5,545	9,927
Panax japonicus subgenome A	271	1,321	1,097	1,100	1,987	5,288	9,927
Panax japonicus subgenome B	286	1,358	1,109	1,006	1,834	5,032	9,927
Panax quinquefolius subgenome A	112	1,475	1,149	1,258	1,671	4,181	9,927
Panax quinquefolius subgenome B	90	1,014	914	1,229	1,580	4,034	9,927
Panax stipuleanatus	849	1,056	939	1,121	2,131	5,434	9,927

* indicates these gene families present in more than one (but all) of the seven genomes/subgenomes (one diploid genome + six tetraploid subgenomes). Numbers from 2 to 6 indicate these genes families present in 2-6 genomes/subgenomes.

Supplementary Table 4. Biased fractionation of the ancestral core-eudicot genes of the four *Panax* species.

Orthologous chromosome**	*Panax stipuleanatus*	*Panax ginseng*		*Panax japonicus*		*Panax quinquefolius*	
		Subgenome A#	Subgenome B	Subgenome A	Subgenome B	Subgenome A	Subgenome B
1	633/731/194*	633/593/44	633/618/24	633/545/56	633/632/68	633/414/33	633/488/31
2	487/655/150	487/539/31	487/553/27	487/486/50	487/530/49	487/378/24	487/400/28
3	846/1198/247	846/1049/44	846/1036/52	846/949/92	846/1005/79	846/713/38	846/640/36
4	638/1662/265	638/1497/56	638/1031/42	638/1346/102	638/1203/87	638/1257/67	638/838/28
5	506/976/209	506/876/51	506/883/48	506/756/62	506/710/71	506/549/34	506/615/26
6	433/761/194	433/658/47	433/650/47	433/564/56	433/625/57	433/531/42	433/325/20
7	486/954/213	486/830/37	486/836/51	486/667/49	486/835/92	486/532/24	486/547/43
8	658/1155/219	658/943/55	658/1029/46	658/828/79	658/968/66	658/666/28	658/689/29
9	536/839/130	536/749/35	536/743/22	536/591/40	536/674/35	536/476/29	536/534/21
10	716/1196/233	716/1064/42	716/1063/49	716/971/90	716/963/67	716/661/30	716/711/30
11	390/723/195	390/563/31	390/667/78	390/535/52	390/502/76	390/424/13	390/468/45
12	545/798/307	545/668/35	545/681/58	545/604/55	545/702/195	545/460/27	545/411/30
Total collinear genes##	21,078	17,411	17,208	16,499	17,165	14,324	13,907
Number of annotated genes###	37,235	34,798	30,262	33,885	34,730	31,641	29,580

Note, *, the three numbers from left to right are core proto-genes, dispensable proto-genes (shared by 4-6 species/subgenome), and dispensable proto-genes (shared by 2-3 species/subgenome), respectively. Information of the core-, dispensable, and unique proto-genes were calculated based on the Supplementary Data 3. **, these orthologous chromosomes are the same as Supplementary Table 1; #, the subgenome A and B are the same as Supplementary Table 1; ##, total number of core and dispensable collinear genes; ###, number of annotated genes in each species or subgenome.

Supplementary Table 5. Biased fractionation of the ancestral core-eudicot genes in the three tetraploid species post the Pg-a duplication.

Orthologous chromosome#	*Panax ginseng*		*Panax japonicus*		*Panax quinquefolius*	
	Subgenome A**	Subgenome B	Subgenome A	Subgenome B	Subgenome A	Subgenome B
1	1173/862/725*	1368/685/549	1173/475/1055	1368/451/1049	1173/575/846	1368/496/935
2	396/1048/955	932/595/443	396/252/1417	932/368/850	396/984/764	932/391/749
3	1240/1250/1089	1450/891/582	1240/556/1580	1450/751/1332	1240/930/1043	1450/460/776
4	1777/1051/783	1490/1073/675	1777/608/1567	1490/717/1831	1777/721/1007	1490/584/940
5	995/891/787	1139/644/499	995/471/835	1139/446/1005	995/536/704	1139/346/467
6	908/733/836	650/807/529	908/345/972	650/434/997	908/544/862	650/509/685
7	980/994/900	1007/819/517	980/504/1125	1007/474/1187	980/638/959	1007/621/964
8	1320/977/624	1476/867/454	1320/710/1118	1476/585/1150	1320/589/793	1476/538/752
9	1370/1019/820	1513/855/529	1370/637/1314	1513/586/1162	1370/548/897	1513/491/921
10	1446/1196/876	1544/937/616	1446/650/1236	1544/693/1174	1446/674/1092	1544/520/853
11	1050/684/715	1002/681/431	1050/430/1014	1002/478/929	1050/466/785	1002/385/630
12	1024/724/580	979/637/397	1024/438/897	979/502/1029	1024/422/583	979/311/706
Total	13679/11429/9690	14550/9491/6221	13679/6076/14130	14550/6485/13695	13679/7627/10335	14550/5652/9378
Total lines	60400/54658/115058					

Note, *, the three numbers from left to right are core proto-genes, dispensable proto-genes, and unique proto-genes, respectively. Information of the core-, dispensable, and unique proto-genes were calculated based on the Supplementary Data 4. #, these orthologous chromosomes are the same as Supplementary Table 1; **, the subgenome A and B are the same as Supplementary Table 1.

Supplementary Table 6. Number of ancestral core-eudicot genes identified in the most correlated regulatory modules of the leaf, root and stem tissues in *Panax stipuleanatus*.

Tissue	Proto-chromosome	Total genes	Transcription factor	Kinase	Synthase	Photosynthesis**
Leaf	Eu1	378	29	12	5	16
	Eu2	278	13	10	11	9
	Eu3	334	22	8	7	19
	Eu4	217	14	8	6	7
	Eu5	286	14	9	12	11
	Eu6	388	25	16	8	10
	Eu7	292	33	13	12	19
	Unassigned*	689	26	27	23	30
Root	Eu1	219	27	6	6	
	Eu2	165	24	9	3	
	Eu3	191	27	3	4	
	Eu4	144	10	7	2	
	Eu5	153	16	7	2	
	Eu6	241	39	7	4	
	Eu7	147	20	14	4	
	Unassigned	351	56	18	3	
Stem	Eu1	116	7	10	0	
	Eu2	77	6	6	0	
	Eu3	88	7	8	1	
	Eu4	82	9	5	0	
	Eu5	72	15	4	0	
	Eu6	92	6	4	3	
	Eu7	67	4	10	1	
	Unassigned	186	14	9	4	

Note, *, these gene cannot be assigned to the seven ancestral core-eudicot chromosomes; **, photosynthesis genes were not included in the statistic of the root and stem tissues.

Supplementary Table 7. Statistical significance of co-expression genes in the seven ancestral core-eudicot chromosomes.

	Eu1*	Eu2	Eu3	Eu4	Eu5	Eu6
Eu2	0.534**					
Eu3	0.891	0.784				
Eu4	0.921	0.804	0.859			
Eu5	0.950	0.535	0.871	0.987		
Eu6	0.684	0.939	0.723	0.460	0.819	
Eu7	0.015	0.159	0.062	0.071	0.163	0.070

*, Eu1-Eu7 are the same as in Figure 1; **, significance is calculated by t-test (two-side).

Supplementary references

1. Marçais, G. & Kingsford, C. A fast, lock-free approach for efficient parallel counting of occurrences of k-mers. *Bioinformatics* **27**, 764–770 (2011).
2. Jiang, P. *et al*. Positive selection driving cytoplasmic genome evolution of the medicinally important ginseng plant genus *Panax*. *Frontiers in Plant Science* **9**, 359 (2018).
3. Chin, C. S. *et al.* Phased diploid genome assembly with single-molecule real-time sequencing. *Nature Methods* **13**, 1050–1054 (2016).
4. Walker, B. J. *et al.* Pilon: An integrated tool for comprehensive microbial variant detection and genome assembly improvement. *PLoS One* **9**, e112963 (2014).
5. Liu, H. et al. SMARTdenovo: a de novo assembler using long noisy reads. *Gigabyte* **2021**, 1–9 (2021).
6. Cagatay Talay, A. & Turgay Altilar, D. RACON: A routing protocol for mobile cognitive radio networks. *Proceedings of the 2009 ACM Workshop on Cognitive Radio Networks* (2009).
7. Burton, J. N. *et al.* Chromosome-scale scaffolding of de novo genome assemblies based on chromatin interactions. *Nature Biotechnology* **31**, 1119–1125 (2013).
8. Burge, C. & Karlin, S. Prediction of complete gene structures in human genomic DNA. *Journal of Molecular Biology* **268**, 78–94 (1997).
9. Stanke, M. & Waack, S. Gene prediction with a hidden Markov model and a new intron submodel. *Bioinformatics* **19**, 215-225 (2003).
10. Majoros, W. H., Pertea, M. & Salzberg, S. L. TigrScan and GlimmerHMM: Two open source ab initio eukaryotic gene-finders. *Bioinformatics* **20**, 2878–2879 (2004).
11. Blanco E, Parra G, Guigó R. Using geneid to identify genes. *Current protocols in bioinformatics* **18**, 1 (2007).
12. Korf, I. Gene finding in novel genomes. *BMC Bioinformatics* **5**, 59 (2004).
13. Keilwagen J, Hartung F, Grau J. GeMoMa: Homology-based gene prediction utilizing intron position conservation and RNA-seq data. *Methods in Molecular Biology* **1962**, 161-177 (2019).
14. Haas, B. J. *et al.* Automated eukaryotic gene structure annotation using evidence modeler and the program to assemble spliced alignments. *Genome Biology* **9,** R7 (2008).
15. Li, H. & Durbin, R. Fast and accurate short read alignment with Burrows-Wheeler transform. *Bioinformatics* **25**, 1754–1760 (2009).
16. Chen, W. *et al*. Whole-genome sequencing and analysis of the Chinese herbal plant *Panax notoginseng*. *Molecular Plant* **10**, 899–902 (2017).
17. Xu, J. *et al*. *Panax ginseng* genome examination for ginsenoside biosynthesis. *Gigascience* **6**, 1-15 (2017).

18. Fan, G. *et al*. The chromosome level genome and genome-wide association study for the agronomic traits of *Panax notoginseng*. *iScience* **23**, 101538 (2020).
19. Jiang, Z. *et al*. The chromosome-level reference genome assembly for *Panax notoginseng* and insights into ginsenoside biosynthesis. *Plant Communication* **2**, 100113 (2021).
20. Yang, Z. *et al.* Chromosomal-scale genome assembly of *Eleutherococcus senticosus* provides insights into chromosome evolution in Araliaceae. *Molecular Ecology Resources* **21**, 2204–2220 (2021).
21. Choi, H. *et al.* Major repeat components covering one-third of the ginseng (*Panax ginseng* C.A. Meyer) genome and evidence for allotetraploidy. *Plant Journal* **77**, 906–916 (2014).
22. Lee, O. R. *et al*. Rapid amplification of four retrotransposon families promoted speciation and genome size expansion in the genus *Panax*. *Scientific Reports* **7**:1–9 (2017).
23. Shim, H. *et al.* Dynamic evolution of *Panax* species. *Genes and Genomics* **43**, 209–215 (2021).
