# Supplementary material for: Reshuffling of the ancestral core-eudicot genome shaped chromatin topology and epigenetic modification in Panax
Source: Nat Commun. 2022 Apr 7;13:1902. doi: 10.1038/s41467-022-29561-5 (PMC8989883; doi:10.1038/s41467-022-29561-5)
Supplement: Supplementary file 3 — Description of Additional Supplementary Files [file 41467_2022_29561_MOESM3_ESM.pdf]

Description of Additional Supplementary Files

File Name: Supplementary Data 1
Description: Number of duplicated ancestral core-eudicot genes in grape and Panax stipuleanatus genomes.

File Name: Supplementary Data 2
Description: Proportion of the gene retention of the six duplicated ancestral core-eudicot chromosomes in Panax stipuleanatus.

File Name: Supplementary Data 3
Description: Collinear matrix of the protein-coding genes of the four Panax species.

File Name: Supplementary Data 4
Description: Matrix of the collinear protein-coding genes of the three tetraploid species.

File Name: Supplementary Data 5
Description: PCA scores of the compartment A/B in Panax stipuleanatus genome.

File Name: Supplementary Data 6
Description: Topological associated domains (TADs) in Panax stipuleanatus genome.

File Name: Supplementary Data 7
Description: Pattenrs of the gene expression and cytosine methylation in all 42 homologous genomic regions.

File Name: Supplementary Data 8
Description: Identification of the CYP450 gene family and their duplciation modes in Panax species.

File Name: Supplementary Data 9
Description: Information of the CYP450 superfamily in Panax stipuleanatus genome.
