# Supplementary material for: Reshuffling of the ancestral core-eudicot genome shaped chromatin topology and epigenetic modification in Panax
Source: Nat Commun. 2022 Apr 7;13:1902. doi: 10.1038/s41467-022-29561-5 (PMC8989883; doi:10.1038/s41467-022-29561-5)
Supplement: Supplementary file 13 — Reporting Summary [file 41467_2022_29561_MOESM13_ESM.pdf]

nature portfolio

Corresponding author(s): Lin-Feng Li, Bao Liu, Yves van de Peer

Last updated by author(s): Mar 14, 2022

Reporting Summary

Nature Portfolio wishes to improve the reproducibility of the work that we publish. This form provides structure for consistency and transparency in reporting. For further information on Nature Portfolio policies, see our Editorial Policies and the Editorial Policy Checklist.

Statistics

For all statistical analyses, confirm that the following items are present in the figure legend, table legend, main text, or Methods section.

n/a	Confirmed	
☐	☒	The exact sample size (n) for each experimental group/condition, given as a discrete number and unit of measurement
☐	☒	A statement on whether measurements were taken from distinct samples or whether the same sample was measured repeatedly
☐	☒	The statistical test(s) used AND whether they are one- or two-sided<br>*Only common tests should be described solely by name; describe more complex techniques in the Methods section.*
☐	☒	A description of all covariates tested
☐	☒	A description of any assumptions or corrections, such as tests of normality and adjustment for multiple comparisons
☐	☒	A full description of the statistical parameters including central tendency (e.g. means) or other basic estimates (e.g. regression coefficient) AND variation (e.g. standard deviation) or associated estimates of uncertainty (e.g. confidence intervals)
☐	☒	For null hypothesis testing, the test statistic (e.g. F, t, r) with confidence intervals, effect sizes, degrees of freedom and P value noted<br>*Give P values as exact values whenever suitable.*
☒	☐	For Bayesian analysis, information on the choice of priors and Markov chain Monte Carlo settings
☒	☐	For hierarchical and complex designs, identification of the appropriate level for tests and full reporting of outcomes
☒	☐	Estimates of effect sizes (e.g. Cohen's d, Pearson's r), indicating how they were calculated

Our web collection on statistics for biologists contains articles on many of the points above.

Software and code

Policy information about availability of computer code

Data collection	All the short Illumina reads were generated based on the platform Illumina Novaseq (Illunima, CA. US). The long reads were generated based on PacBio RSII platform (PacBio, USA) and Nanopore platform (Nanopore, UK).Karyotypes of the four species were visualized using OLYMPUS BX53 (Olympus Corporation, Japan).
Data analysis	Data analyses were performed using the publicly available softwares. Details of these softwares were described in the Methods and Supplementary Information, such as PAML (v4.9h) package, bowtie2 (version 2.2.3), and ShortStack (version 3.8.3).

For manuscripts utilizing custom algorithms or software that are central to the research but not yet described in published literature, software must be made available to editors and reviewers. We strongly encourage code deposition in a community repository (e.g. GitHub). See the Nature Portfolio guidelines for submitting code & software for further information.

Data

Policy information about availability of data

All manuscripts must include a data availability statement. This statement should provide the following information, where applicable:

- Accession codes, unique identifiers, or web links for publicly available datasets
- A description of any restrictions on data availability
- For clinical datasets or third party data, please ensure that the statement adheres to our policy

All sequence read assemblies have been deposited into the National Center for Biotechnology Information (https://www.ncbi.nlm.nih.gov) under the BioProject number PRJNA752920 and National Genomics Data Center (https://ngdc.cncb.ac.cn) under the project number PRJCA006678.The whole genome sequence data reported in this paper have been deposited in the Genome Warehouse in National Genomics Data Center79,80, Beijing Institute of Genomics, Chinese Academy of Sciences/China National Center for Bioinformation, under accession number GWHBEIH00000000, GWHBEIJ00000000, GWHBEIL00000000 and GWHBEIR00000000

that is publicly accessible at https://ngdc.cncb.ac.cn/gwh.

Field-specific reporting

Please select the one below that is the best fit for your research. If you are not sure, read the appropriate sections before making your selection.

- [ ] Life sciences
- [ ] Behavioural & social sciences
- [x] Ecological, evolutionary & environmental sciences

For a reference copy of the document with all sections, see nature.com/documents/nr-reporting-summary-flat.pdf

Ecological, evolutionary & environmental sciences study design

All studies must disclose on these points even when the disclosure is negative.

Study description	We assembled the reference genomes of four Panax species and reconstructed the evolutionary history of core-eudicot genome inthe four extant species. Then, we addressed how this reshuffling of ancestral core-eudicot genome affect the chromatin topology and epigenetic modification in the extant Panax genomes.
Research sample	Samples of the four Panax species were collected from the field and grow in greenhouse. For the genome assembly, only one accession of each species was selected. For the gene expression and metabolic comparisons, three accessions were randomly selected.
Sampling strategy	For the gene expression and metabolic comparisons, three biological replicates are enough for this kind of data analyses.
Data collection	All the sequence data were generated from sequencing platforms, such Illumina Novaseq (Illumina, CA, US),PacBio RSII (PacBio, USA) and Nanopore (Nanopore, UK).
Timing and spatial scale	All these samples were collected during 2018-2019. Data analyses were performed during 2019-2020. We started most of the data analyses after we obtained the genome assemblies of the four Panax species.
Data exclusions	No original data were excluded from this study.
Reproducibility	Data collections and analyses of these biological replicates were performed at the same time. Parameters for the data analyses were also provided in the Methods and Supplementary Information.
Randomization	All samples were collected randomly.
Blinding	We randomly selected the samples and subjected to do the sequencing.

Did the study involve field work? [ ] Yes [x] No

Reporting for specific materials, systems and methods

We require information from authors about some types of materials, experimental systems and methods used in many studies. Here, indicate whether each material, system or method listed is relevant to your study. If you are not sure if a list item applies to your research, read the appropriate section before selecting a response.

Materials & experimental systems

n/a	Involved in the study
[x]	[ ] Antibodies
[x]	[ ] Eukaryotic cell lines
[x]	[ ] Palaeontology and archaeology
[x]	[ ] Animals and other organisms
[x]	[ ] Human research participants
[x]	[ ] Clinical data
[x]	[ ] Dual use research of concern

Methods

n/a	Involved in the study
[x]	[ ] ChIP-seq
[x]	[ ] Flow cytometry
[x]	[ ] MRI-based neuroimaging
